# Metabolic plasticity enables lifestyle transitions of *Porphyromonas gingivalis*

**DOI:** 10.1038/s41522-021-00217-4

**Published:** 2021-05-24

**Authors:** M. Fata Moradali, Mary E. Davey

**Affiliations:** 1 Department of Oral Biology, College of Dentistry, University of Florida, Gainesville, FL USA; 2 Present Address: Department of Oral Immunology and Infectious Diseases, University of Louisville, School of Dentistry, Room 355 B, Louisville, KY USA

**Keywords:** Plaque, Pathogens

## Abstract

Our understanding of how the oral anaerobe *Porphyromonas gingivalis* can persist below the gum line, induce ecological changes, and promote polymicrobial infections remains limited. *P. gingivalis* has long been described as a highly proteolytic and asaccharolytic pathogen that utilizes protein substrates as the main source for energy production and proliferation. Here, we report that *P. gingivalis* displays a metabolic plasticity that enables the exploitation of non-proteinaceous substrates, specifically the monocarboxylates pyruvate and lactate, as well as human serum components, for colonization and biofilm formation. We show that anabolism of carbohydrates from pyruvate is powered by catabolism of amino acids. Concomitantly, the expression of fimbrial adhesion is upregulated, leading to the enhancement of biofilm formation, stimulation of multispecies biofilm development, and increase of colonization and invasion of the primary gingival epithelial cells by *P. gingivalis*. These studies provide the first glimpse into the metabolic plasticity of *P. gingivalis* and its adaptation to the nutritional condition of the host niche. Our findings support the model that in response to specific nutritional parameters, *P. gingivalis* has the potential to promote host colonization and development of a pathogenic community.

## Introduction

An orchestrated series of dysbiotic polymicrobial interactions can promote the colonization of a pathogenic microbial community below the gum line, leading to periodontitis, an infection-driven inflammatory disease of the periodontal tissues and alveolar bone, imposing adverse systemic effects on human health^1–5^. Various etiological models suggest that the Gram-negative anaerobe *Porphyromonas gingivalis* may play a central role in the onset of periodontitis via orchestrating a pronounced ecophysiological change below the gingival margin^6,7^; however, the mechanistic basis of such a microbial shift has not been elucidated. *P. gingivalis* can persist in the subgingival ecosystem adjacent to the epithelium, even in the absence of periodontitis in an otherwise healthy individual^8–11^; hence, it is speculated that an unknown ecophysiological condition allows *P. gingivalis* strains to transition toward a pathogenic state, and to produce an array of pathogenicity factors, including the type IX secretion system (T9SS), proteases, and fimbrial adhesions, that promote bacterial proliferation, persistence, and invasion^6,12,13^. Therefore, understanding the environmental factors that affect the physiological state of *P. gingivalis* is crucial.

Historically, *P. gingivalis* has been known as a highly proteolytic and asaccharolytic anaerobe that secretes potent proteases known as gingipains via the T9SS constituted by at least ten interacting proteins, including PorK/L/M/N/P/Q/T/U/V/W and SprA (also termed Sov), that span the cell envelope^14^. T9SS and gingipains are essential determinants of *P. gingivalis* virulence and are necessary for breaking down the host proteinaceous substrates into small peptides, which are then transported into the cytoplasm for fermentation and energy production; intriguingly, the availability of free amino acids and carbohydrates does not meet this requirement^15–18^.

Our previous work demonstrated that *P. gingivalis* strain 381 (a highly fimbriated and robust biofilm-forming strain) is capable of switching between sessile growth and surface translocation, when sandwiched between two surfaces^19^. Although the T9SS and secreted proteases were found necessary for biofilm-to-migration transition, such a lifestyle transition was also dictated by nutritional stimuli and signals. As a proof of concept, specific substrates including the key monocarboxylates pyruvate and lactate, along with bovine serum albumin (BSA) were tested and found to impede the transition of *P. gingivalis* to a migration state. Instead, these nutrients promoted biofilm development under a migration-inducing condition through a yet unknown mechanism^19^. Pyruvate and lactate are not only key carbon and energy sources for living organisms, but also have other biological functions. For instance, sodium pyruvate is an efficient peroxide scavenger that confers protection to host cells and oral streptococci by detoxifying hydrogen peroxide in the environment^20–27^. In the aerobic and saccharolytic lifestyle, pyruvate is produced from glucose metabolism during glycolysis that eventually enters the tricarboxylic acid (TCA) cycle and the electron transfer chain, while it can be reduced to lactate under anaerobic conditions^28^. However, except for anaerobic sulfate reducing bacteria (e.g., *Desulfovibrio*) that couple lactate oxidation to sulfate reduction, metabolism of pyruvate, and lactate in obligately anaerobic and asaccharolytic organisms has largely remained unknown. The increase of pyruvate and lactate in the intracellular and extracellular milieus of human cells and serum have been reported to be due to a variety of factors, including microbial fermentation^5,29^, inflammation^5,30^, physiological diseases^31–33^, the Warburg effect^34,35^, stresses^26,36,37^, and diets^38–40^; yet how the availability of pyruvate and lactate and serum components impacts the physiology of *P. gingivalis* and other oral residents is not clear.

In this work, we report that *P. gingivalis* can utilize pyruvate and lactate in the presence of human serum. Our data show that when combined with human serum, exogenous pyruvate is an important energy substrate for *P. gingivalis* that affects not only central carbon metabolism, but also the expression of fimbrial adhesion, a requirement for surface colonization, biofilm development, and invasion. Overall, our findings indicate that such plasticity in nutrient utilization allows the phenotypic adaptation of *P. gingivalis* to the nutritional parameters of the host environment.

## Results

### The utilization of pyruvate and lactate requires metabolic coupling with amino acids fermentation

**Fig. 1:**
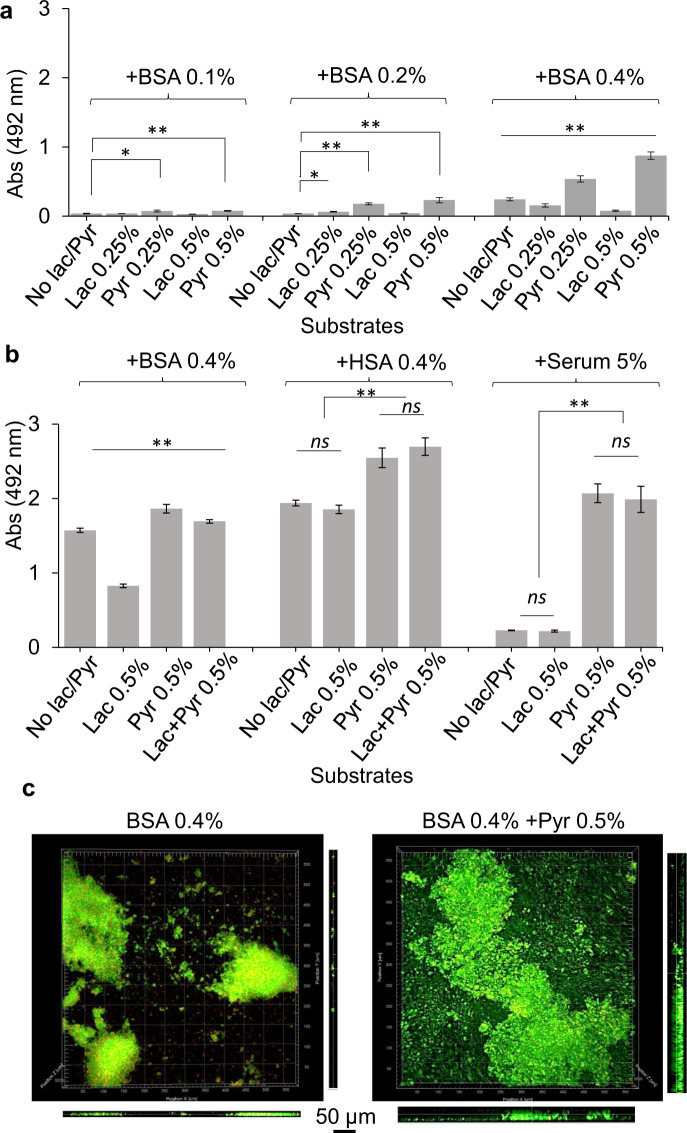
Synergistic effects of the metabolism of pyruvate, lactate, and serum components on the growth and biofilm formation of *P. gingivalis*. **a** Biofilm assessments show that the utilization of BSA enhances *P. gingivalis* biofilm in a concentration-dependent manner. Pyruvate and lactate as sole carbon and energy sources cannot support *P. gingivalis* growth and biofilm formation, while the combination of BSA-pyruvate and BSA-lactate, respectively, promotes and inhibits biofilm formation. **b** Pyruvate enhances biofilm development in combination with human serum albumin (HSA) or human serum, but lactate is not inhibitive to biofilm growth neither alone nor in combination with pyruvate. **c** Images of 24-h biofilms were acquired by CLSM. Top and side views show that pyruvate significantly promotes surface attachment and biofilm development by *P. gingivalis*. Graphs represent mean biomass of biofilms ± SE (two biological replicates, in each *n* = 3) at 48 h, as determined by safranin staining. Biofilm biomasses were normally distributed (Shapiro–Wilk test: *p* > 0.05), and thus analyzed using ANOVA test. Asterisks indicate pairs of significantly different values (post hoc Tukey’s HSD test: *p < 0.05; **p < 0.01; ns not significant).

This study sought to understand how pyruvate, lactate, and serum components may impact the physiology of *P. gingivalis* and the transition between sessile growth and surface translocation^19^. First, our preliminary assessment using static liquid cultures showed that *P. gingivali*s strain 381 can efficiently utilize BSA protein for proliferation, but BSA-only medium progressively induced the auto-aggregation and settling of *P. gingivalis* cells over time when compared with a nutrient-rich laboratory medium, such as TSBHK (Supplementary Fig. 1). As expected, pyruvate- or lactate-only medium could not support *P. gingivalis* proliferation. After being combined with BSA protein, pyruvate enhanced cell proliferation while lactate reduced it (Supplementary Fig. 1). Consistent with these findings, our biofilm assessments showed that the combination of BSA and pyruvate could enhance biofilm formation, while biofilm formation was inhibited in the presence of lactate in a concentration-dependent manner (Fig. 1a). Similar assessment under microaerophilic condition showed that the effects of pyruvate and lactate on biofilm formation are similar to anaerobic condition, despite the limited biofilm-forming capacity under microaerophilic condition (Supplementary Fig. 2a). We then assessed the impact of pyruvate and lactate on biofilm formation in a human serum albumin (HSA)-based medium. Similarly, pyruvate enhanced biofilm development when combined with HSA by 1.3 times; however, lactate did not impose a significant inhibitive effect on biofilm growth in the HSA-based medium (Fig. 1b). Further, we used a human serum-only medium to better simulate subgingival conditions. Interestingly, *P. gingivalis* strain 381 could not establish a robust biofilm in human serum-only medium, while supplementation with pyruvate, but not lactate, remarkably increased biofilm development by ten times (Fig. 1b). Visualization of biofilms by confocal laser scanning microscopy (CLSM) validated our findings, demonstrating that *P. gingivalis* has a higher capacity of surface attachment and biofilm formation in the presence of pyruvate (Fig. 1c and Supplementary Fig. 2b).

**Fig. 2:**
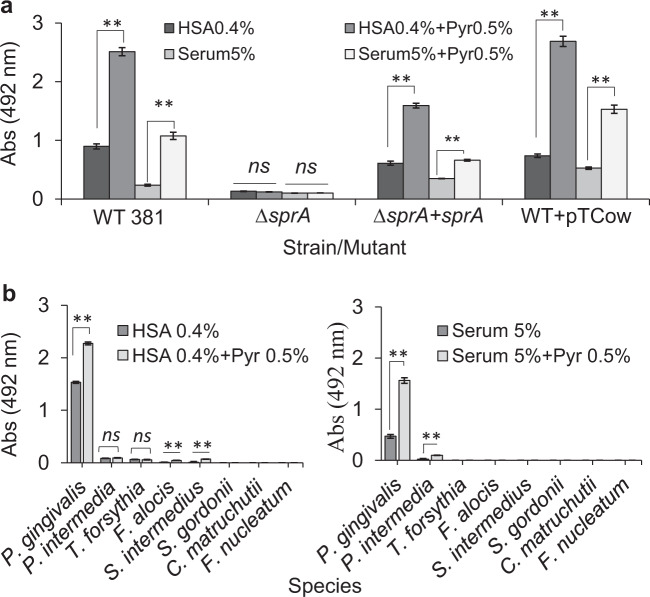
Synergistic effects of the metabolism of pyruvate, lactate, and serum components on the biofilm formation of *∆sprA* mutant, that lacks the essential translocon of the T9SS, and other oral pathogens. **a** Biofilm assay shows *P. gingivalis* ∆*sprA* mutant cannot efficiently utilize serum components for biofilm development; this mutant is not responsive to pyruvate addition. But, upon *in trans* complementation with *sprA*, biofilm development was restored by the mutant, indicating there must be a metabolic coupling system to utilize protein components and pyruvate whose synergistic effect enhances metabolic flux towards biofilm development. **b** Mono-species biofilm assays show that among tested oral pathogens, only *P. gingivalis* effectively benefits from the synergistic effects of concomitant utilization of pyruvate and serum components for enhancing biofilm. Statistically, *P. intermedia*, *S. intermedius*, and *F. alocis* positively responds to pyruvate addition but at very low levels. Graphs represent mean biomass of biofilms ± SE (two biological replicates, in each *n* = 3) at 48 h, as determined by safranin staining. Biofilm biomasses were normally distributed (Shapiro–Wilk test: *p* > 0.05), and thus analyzed using ANOVA test. Asterisks indicate pairs of significantly different values (post hoc Tukey’s HSD test: *p < 0.05; **p < 0.01; ns not significant).

It is noteworthy to mention that a specific pyruvate transporter has not been identified in *P. gingivalis*. Given that the effects of pyruvate on *P. gingivalis* physiology and growth were only observed when proteinaceous components were provided, and the T9SS and its cargo proteases are central to the generation of peptide substrates, we hypothesized that a reduction in the availability of oligopeptides via disruption of T9SS function would negatively impact the metabolic coupling of pyruvate and proteinaceous components. To test this hypothesis, we applied a *P. gingivalis* ∆*sprA* mutant, a strain that lacks the essential translocon of the T9SS^14,19,41^. Despite having a limited growth capability, the ∆*sprA* mutant displayed a biofilm phenotype that enabled us to test the hypothesis above. Biofilm assessments showed that *P. gingivalis* ∆*sprA* was not responsive to pyruvate addition, while in trans complementation with *sprA* (*P. gingivalis* ∆*sprA*+pTCOW::*sprA*) restored the pyruvate-induced phenotypic change and significantly increased biofilm formation (Fig. 2a), supporting our notion that *P. gingivalis* may possess an as yet unreported biofilm-enhancing metabolic system that responds to certain nutritional factors that may also impede the transition toward surface translocation under migration-inducing condition^19^.

To test whether such a proposed metabolic coupling system exists in other biofilm-forming strains of *P. gingivalis*, we assessed the impact of the same growth and nutritional conditions on other fimbriated strains W50 and ATCC 33277. Our data showed that the addition of pyruvate to HSA or human serum could increase the biofilms of strains W50 and ATCC 33277 by up to 3.3 and 1.6 times, respectively (Supplementary Fig. 3). Moreover, we assessed the impact of serum components and/or pyruvate on mono-species biofilms of other oral bacteria, including *Prevotella intermedia*, *Tannerella forsythia*, *Filifactor alocis*, *Streptococcus gordonii*, *Staphylococcus* *intermedius, Corynebacterium matruchotii*, and *Fusobacterium nucleatum*. Our data showed that in general these bacteria are unable to efficiently harness nutritional values of serum components and pyruvate for promoting biofilm formation, when compared with *P. gingivalis* (Fig. 2b). However, to a much less degree, *P. intermedia*, *S. intermedius*, and *F. alocis* could positively respond to pyruvate addition. Overall, these findings indicated that *P. gingivalis* has evolved with a yet unreported metabolic plasticity that enables efficient utilization of host-driven non-proteinaceous substrates, as well as serum components toward enhancing surface colonization and biofilm development.

### Metabolic plasticity controls differential expression of pathogenicity factors in *P. gingivalis*

**Fig. 3:**
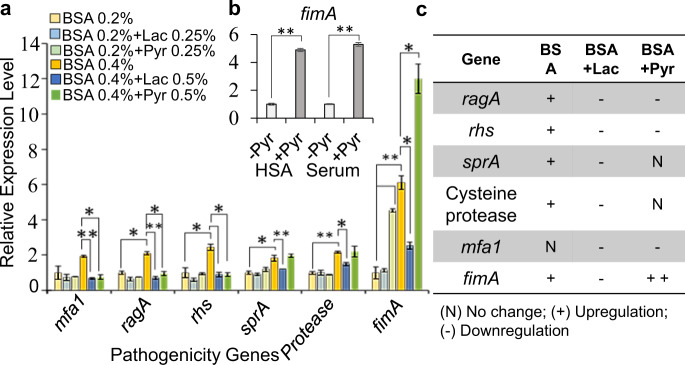
Various pathogenicity genes are differentially expressed in response to the availability of specific energy substrates, i.e., pyruvate or lactate and/or serum components. **a** RT-qPCR analysis and relative expression levels shows that, consistent with biofilm results, BSA utilization increases the expression of *fimA* in a concentration-dependent manner; the addition of pyruvate in the BSA-based medium remarkably increases the expression of *fimA*. In addition, the provision of higher concentrations of BSA correlates positively with the expression of those pathogenicity genes involved in the degradation of protein substrates and oligopeptide uptake. Exogenous lactate significantly decreases the expression of *fimA* and other pathogenicity genes when compared with untreated controls, leading to decreased biofilm formation. **b** The addition of pyruvate in HSA or human serum also significantly increases the expression of *fimA*. **c** Table summarizes RT-qPCR results and shows the correlation of utilized energy substrates and expression of pathogenicity genes. Graphs represent mean expression ± SE (two biological replicates, in each *n* = 3). Asterisks indicate pairs of significantly different values (post hoc Tukey’s HSD test: *p < 0.05; **p < 0.01).

To determine how the given metabolic coupling system may contribute to the regulation of gene expression, including biofilm-formation-related genes, we performed quantitative reverse transcription PCR (RT-qPCR) analysis on RNA samples extracted from biofilms grown in BSA-based medium with and without adding pyruvate or lactate. Figure 3a represents the relative expression level of specific key virulence factors in response to a given substrate. The comparison of relative gene expression showed that only the *fimA* gene encoding the main component of major fimbriae was greatly changed among various target genes; *fimA* expression was significantly upregulated in response to pyruvate addition in the BSA-, HSA-, or human serum-based media (Fig. 3a, b). On the other hand, pyruvate addition significantly decreased the expression of *ragA* (the most abundant oligopeptide transporter in the outer membrane), *rhs* (a major component of contact-dependent inhibition module), and *mfa1* (the major protein subunit of minor fimbriae); the expression of *sprA* (the main constitutive component of T9SS) remained unchanged. Further, we determined that higher concentration of BSA in the medium has a positive correlation with the expression of *sprA*, *ragA*, *rhs*, and a cysteine protease (PGN_0508), while the expression of *mfa1* remained unchanged (Fig. 3a, c). Consistent with the biofilm data showing that lactate has an inhibitive effect on biofilm formation, the addition of lactate to the BSA-based medium could downregulate the expression of all targeted genes, including *fimA* expression (by 2.3 times), when compared with the BSA-only medium (Fig. 3a, c). Overall, these findings revealed that specific pathogenicity factors are differentially expressed in response to the availability of key biologically relevant substrates; indeed, the expression of *fimA* is greatly affected by the availability of pyruvate and lactate. FimA fimbria is the main mediator of *P. gingivalis* colonization and attachment to microbial counterparts and host cells^42–50^; hence, RT-qPCR data were consistent with the observed phenotypic changes in *P. gingivalis*, including enhanced auto-aggregation, surface attachment, and biofilm formation.

### Metabolic plasticity controls anaerobic redox homeostasis and affects fimbria expression

**Fig. 4:**
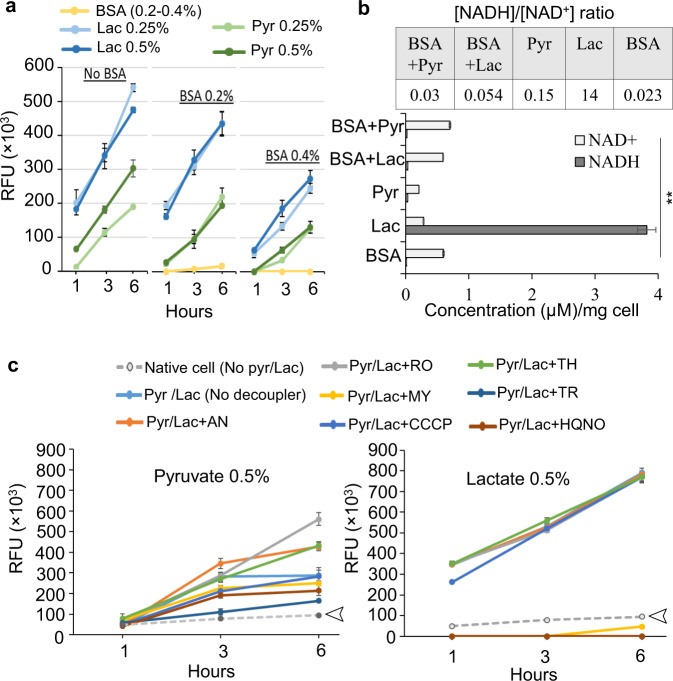
The impact of proteinaceous substrates, pyruvate, and lactate on the redox status of *P. gingivalis*. **a** Resazurin fluorometric (RF) assay indicates that exogenous lactate and pyruvate greatly change cellular redox status, but BSA utilization does not; concomitant utilization of BSA and pyruvate or lactate effectively controls redox changes. **b** Quantification of [NADH]/[NAD^+^] cofactors shows that the metabolism of BSA enhances cellular accumulation of [NAD^+^] when compared with the utilization of exogenous pyruvate that reduces it. Lactate metabolism remarkably favored cellular accumulation of [NADH] and the increase of [NADH]/[NAD^+^] ratio, while it is significantly lowered upon BSA addition. **c** The use of a series of chemical uncouplers in RF assay to shed light on the bioenergetics system of *P. gingivalis* and the effects of pyruvate and lactate on it. RF assays over 6-h incubation period indicated that the uncouplers HQNO (a potent inhibitor of quinone oxidation at the Na^+^–NQR complex in bacteria), TR (a calmodulin antagonist and type II NADH oxidoreductase (NDH-2) inhibitor in *M. tuberculosis*), and MY (a Q_o_ site inhibitor at cytochrome *b*) have strong inhibitory effects on lactate-induced redox changes upon decoupling electron transport system from lactate metabolism while others, including AN, RO, CCCP, and TH did not show inhibitory effects. The uncouplers HQNO, TR, and MY were less inhibitive to pyruvate-induced redox change. Arrows indicate the basal redox status of the cells in the absence of any treatment. The data in graphs represent the means ± SD error for biological replicates (*n* = 4), and asterisk in the bar graph indicates significantly different values (post hoc Tukey’s HSD test: **p < 0.01). RFU relative fluorescence units, BSA bovine serum albumin, Pyr pyruvate, Lac lactate, HQNO 2-heptyl-4-hydroxyquinoline N-oxide, TR trifluoperazine, MY myxothiazol, AN antimycin A, RO rotenone, CCCP carbonyl cyanide m-chlorophenyl hydrazine, TH thenoyltrifluoroaceton.

In living organisms, the conversion of pyruvate to lactate or to other metabolic intermediates is concomitant with alterations in the ratio of [NADH]/[NAD^+^] cofactors and cellular redox status, which in turn affects the dynamics of cellular metabolism, energy production, and numerous biological processes^51,52^. Here, we used a resazurin fluorometric (RF) assay to assess the redox status of *P. gingivalis*, when pyruvate, lactate, and serum components are concomitantly utilized. In this assay, nonfluorescent resazurin molecules (an oxidation–reduction indicator) are irreversibly converted to highly fluorescent resorufin molecules when coupled with the oxidation of NADH, and this conversion is used to infer the metabolism of different substrates. The NADH turnover and conversion of resazurin to resorufin is measured as the relative fluorescent units (RFU)^53^. Upon the addition of different substrates to the cell suspensions, redox changes were fluorometrically measured over a 6-h incubation period. RFU calculation showed that the conversion of resazurin to resorufin was trivial upon the utilization of various concentrations of BSA, indicating no significant change was induced in the redox status of *P. gingivalis* upon BSA utilization, but exogenous lactate and pyruvate greatly altered cellular redox status, resulting in a high level of fluorescence yield (Fig. 4a). Interestingly, the pyruvate/lactate-mediated redox change was greatly alleviated upon BSA addition in a concentration-dependent manner (Fig. 4a). Also, the quantification of cellular concentration of [NADH] and [NAD^+^] showed that the utilization of BSA increases the cellular accumulation of [NAD^+^], while it was significantly reduced upon pyruvate availability. On the other hand, lactate utilization remarkably favored the cellular accumulation of [NADH] and significantly increased [NADH]/[NAD^+^] ratio, while this reducing power was significantly lowered upon BSA addition (Fig. 4b).

To shed light on the unknown bioenergetic system of *P. gingivalis* and its possible engagement with the metabolism of pyruvate and lactate, we combined the RF assay with the application of a series of chemical uncouplers that show inhibitory activity at specific sites of electron transport systems^54^. Among applied uncouplers (Fig. 4c), 2-heptyl-4-hydroxyquinoline N-oxide (HQNO; a potent inhibitor of quinone oxidation at the Na^+^–NQR complex in bacteria), trifluoperazine (TR; a calmodulin antagonist and type II NADH oxidoreductase (NDH-2) inhibitor in *Mycobacterium tuberculosis*), and myxothiazol (MY; a Q_o_ site inhibitor at cytochrome *b*) strongly inhibited the lactate-induced redox change; others including antimycin A, rotenone, carbonyl cyanide 3-chlorophenyl hydrazone, or thenoyltrifluoroacetone did not impact redox changes (Fig. 4c). On the other hand, HQNO, TR, and MY were less inhibitive to the pyruvate-induced redox change compared to the lactate-induced redox change. Overall, the effectiveness of uncouplers suggests that the central electron transport system of *P. gingivalis* is composed of the Na^+^-translocating NADH:quinone oxidoreductase (Na^+^–NQR; PGN_0116-0118), NADH oxidoreductases (possibly including PGN_0285, an uncharacterized NAD(FAD)-dependent dehydrogenase), and cytochrome *bd* ubiquinol oxidases (PGN_1041-42), which also mediate the engagement with the metabolism of pyruvate and lactate (Fig. 4c; see “Discussion” for the proposed model). To validate the inhibitory effects of selected uncouplers on the ecological performance of *P. gingivalis*, we assessed their effects on biofilm formation and surface translocation. Our data showed that HQNO, TR, and MY have potent inhibitory effects on the biofilm development and surface translocation of *P. gingivalis* (Supplementary Fig. 4a, b).

Given that the availability of pyruvate and lactate impacts the expression levels of specific pathogenicity genes and also their metabolism is linked to the bioenergetic system, we hypothesized that the expression of specific pathogenicity genes may occur in coordination with bioenergetic status. To test this hypothesis, we performed RT-qPCR analysis on RNA samples isolated from HQNO (10 μg/ml)-treated cells to compare with untreated control cells. The quantification of relative gene expression showed that even though HQNO reduces the expression levels of *ragA*, *rhs*, *mfa1*, *sprA*, and *nqr* genes by up to two times, *fimA* expression was downregulated by almost five times (Supplementary Fig. 4c, d), indicating that *fimA* expression is highly regulated in coordination with the bioenergetic status of the cells. Overall, our findings provided the first insight into the interplay of amino acids fermentation coupled with pyruvate and lactate metabolism along with elements of the bioenergetic system of *P. gingivalis* that may control both cellular redox homeostasis and the expression of specific pathogenicity genes.

### Pyruvate availability rewires carbon flux toward de novo biosynthesis of carbohydrates and anabolic processes in *P. gingivalis*

**Fig. 5:**
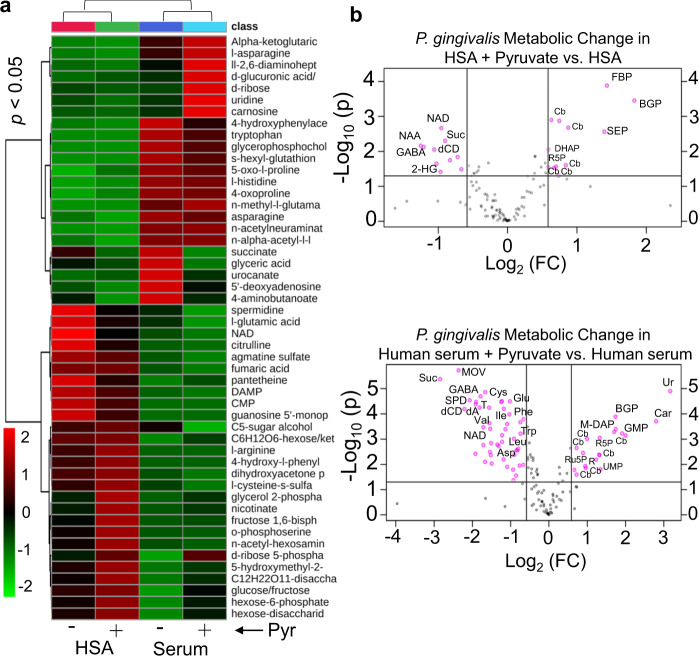
Statistical analysis of metabolic changes among all groups of *P. gingivalis* biofilm cells grown in HSA or human serum with and without adding pyruvate. **a** Heatmap represents multigroup analysis for the metabolites of all groups that are found as differentially produced upon one-way ANOVA followed by post hoc analysis (*p* value <0.05, *n* = 3) and variable importance in projection (VIP) values >1.0. **b** Two-group volcano plot analysis of metabolic changes in HSA or human serum with and without pyruvate. The points in each plot that satisfy the condition *p* value <0.05 (*t* test, *n* = 3) and fold change ≥ 1.5 appear in pink color, whereas the others that appear in gray are not significant. OAA oxaloacetate, KG α-ketoglutarate, Lac lactate, PEP phosphoenolpyruvate, ETC electron transport chain, BGP glycerol 2-phosphate, FBP fructose-1,6-bisphosphate, SEP O-phosphoserine, Cb carbohydrate derivative, NAD nicotinamide adenine dinucleotide, Suc succinate, GABA 4-aminobutyric acid or 4-aminobutanoate, NAA N-acetylaspartic acid, 2-HG 2-hydroxyglutarate, DHAP dihydroxyacetone phosphate, Ur uridine, MOV 3-methyl-2-oxovaleric acid, dCD deoxycytidine, SPD spermidine, dA deoxyadenosine, T thymine, G6P glucose-6-phosphate, M-DAP LL-2,6-diaminopimelic acid, R ribose, R5P ribose-5-phosphate, Ru5P ribulose-5-phosphate, UMP uridine monophosphate, Cys cysteine, Glu glutamic acid, Ile isoleucine, Val valine, Trp tryptophan, Leu leucine, Asp aspartic acid.

Given that pyruvate plays a central role in several metabolic pathways in all organisms, and our findings showed that it greatly enhances fimbria-mediated attachment and biofilm formation, we aimed at revealing the impact of concomitant utilization of pyruvate and serum components on metabolic status of *P. gingivalis* during biofilm growth. To this end, we performed a global metabolomic analysis on the metabolome of the biofilm cells grown in HSA or human serum with and without pyruvate addition. For multigroup analysis, one-way analysis of variance (ANOVA) followed by post hoc analysis (*p* value <0.05) in addition to variable importance in projection values >1.0 revealed that 74 metabolites of canonical pathways (out of a total of 103 metabolites) were differentially produced in biofilm-growing cells in response to the given substrates (Supplementary Tables 1 and 2). The heatmap and principal component analysis of a multigroup comparison are shown in Fig. 5a and Supplementary Fig. 5a. Since the genome sequences of *P. gingivalis* strains are available, canonical metabolic networks associated with pyruvate metabolism was predicted and visualized, using MetScape visualization tool (Supplementary Fig. 5b). Multigroup analysis showed that metabolic profile of biofilm cells greatly varies between the HSA- and human serum-based media. Upon metabolizing HSA, a large number of amino acids and their derivatives, e.g., citrulline, agmatine sulfate, glutamic acid, and amidino-l-aspartate were produced in the cells. Compared to the utilization of human serum, HSA utilization increased the synthesis of the nucleoside monophosphates, including guanosine monophosphate and cytidine monophosphate, NAD cofactor, and pantetheine (an intermediate in the production of coenzyme A). On the other hand, human serum significantly increased cellular concentration of α-ketoglutaric acid (or 2-oxoglutaric acid), the key intermediate of the TCA cycle that plays as a metabolic hub at the intersection between the carbon and nitrogen metabolic pathways (Fig. 5a and Supplementary Fig. 6a). GABA (or 4-aminobutanoate) and succinate that are important intermediates of the GABA shunt, a bypass route from α-ketoglutarate, were found at a higher level upon human serum utilization when compared with the HSA medium; histidine, urocanate (a histidine-derived metabolite), aspartate, asparagine, tryptophan, methyl-l-glutamate, hydroxyphenylacetate, and n-acetylneuraminate were also higher in the biofilm cells grown in human serum medium (Fig. 5a and Supplementary Fig. 5b).

For two-group analysis, i.e., HSA or human serum with and without adding pyruvate, a student’s *t* test and fold-change (FC) analysis were executed and statistically significant metabolites (FC threshold 1.5 and *p* value <0.05) were presented in the volcanic plots (Fig. 5b). Addition of pyruvate to the HSA- and human serum-based media significantly altered a total of 23 and 66 metabolites in the biofilm cells, respectively, when compared to their respective untreated media (Supplementary Table 3 and Supplementary Fig. 6b). Analysis of these metabolites within canonical metabolic pathways showed that, in general, metabolic changes induced by exogenous pyruvate in both groups follow a similar pattern; thus, in both groups, pyruvate reduced GABA shunt metabolites, including GABA and succinate, as well as NAD cofactor, which was consistent with our data of the quantification of [NADH]/[NAD^+^] cofactors. On the other hand, pyruvate increased cellular accumulation of glucose-6-phosphate, hexoses, trioses, ribose, ribose-5-phosphate, ribulose-5-phosphate, and fructose-1, 6-bisphosphate. These are metabolic intermediates originating from the pentose phosphate pathway (the PPP; also known as the phosphogluconate pathway or the hexose monophosphate shunt) and gluconeogenesis (Fig. 5b). These pathways are critical metabolic foundations for anabolic processes, including biosynthesis of macromolecules, nucleic acids, and energy molecules, e.g., NADH and NADP^55–57^.

Given that our data showed that n-acetylneuraminate is also a pronounced metabolite in the biofilm cells when grown in the human serum medium, and it is a predominant sialic acid in human serum that can serve as a nitrogen/carbon source via entering the PPP^58^, we assessed metabolic plasticity of *P. gingivalis* in the utilization of free n-acetylneuraminate for energy production and biofilm formation. Biofilm assays showed that *P. gingivalis* can form biofilm upon the utilization of free n-acetylneuraminate as a sole nitrogen/carbon and energy source (Supplementary Fig. 7), and concomitant utilization of n-acetylneuraminate and pyruvate significantly enhanced biofilm formation. However, mucin-associated n-acetylneuraminate could not support *P. gingivalis* biofilm formation, and neither did pyruvate when combined with mucin (Supplementary Fig. 7). These data indicate that despite the fact that *P. gingivalis* has evolved with a sialidase (PGN_1608), this enzyme does not act extracellularly; further, bioinformatics databases predicted the presence of a transmembrane domain in its structure that may guide its localization in the inner membrane. Overall, these findings support our notion that exogenous pyruvate is tied to the metabolism of serum components toward the enhancement of the PPP and biosynthetic processes that are required during the biofilm development (see “Discussion” for the proposed model).

### Concomitant utilization of pyruvate and serum components promotes multispecies biofilms, colonization, and invasion of host cells by *P. gingivalis*

**Fig. 6:**
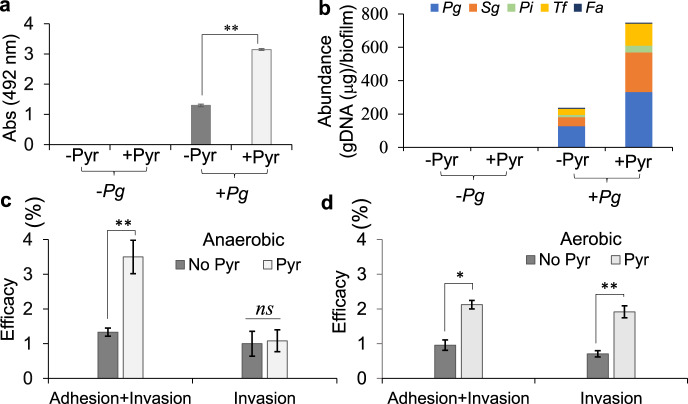
The impact of the co-utilization of pyruvate and human serum by *P. gingivalis* on development of multispecies biofilms, host colonization, and invasion. **a** Multispecies biofilm assay shows that *P. gingivalis* is necessary for the formation of a heterotypic community composed of *S. gordonii, T. forsythia, P. intermedia*, and *F. alocis* when human serum (5%) is used; pyruvate addition (0.5%) augments the biomass of the heterotypic biofilm. **b** Graph shows relative abundance of each species in each mixed biofilm that is calculated on the basis of the quantification of the genomic DNA of individual population using qPCR and 16S ribosomal RNA primers. **c**, **d** Graphs show the efficacy of colonization and invasion of the primary HGEP cells by *P. gingivalis* (MOI of 100 and incubated for 1 h) when grown in human serum with and without adding pyruvate, incubated under anaerobic (**c**) or aerobic (**d**) conditions. Pyruvate availability significantly increases the adherence of *P. gingivalis* to the primary HGEP under both aerobic and anaerobic conditions; but invasion was augmented when the interaction was aerobically incubated. Biofilm graph represents the mean ± SE (two biological replicates, in each *n* = 3) of biomass incubated for 48 h. Obtained data were normally distributed (Shapiro–Wilk test: *p* > 0.05), and thus analyzed using ANOVA test. Asterisks indicate pairs of significantly different values (post hoc Tukey’s HSD test: **p < 0.01). Values in graphs are shown as the mean ± SE of CFU obtained from two independent experiments, each with four replicates, and were analyzed with a Student’s *t* test (*p < 0.05; **p < 0.01; ns nonsignificant). *Pg* *P. gingivalis* 381, *Sg* *S. gordonii* DL-1, *Pi* *P. intermedia* strain 17, *Fa* *F. alocis* ATCC 35896, *Tf* *T. forsythia* ATCC 43037.

Indeed, fimbrial adhesions mediate co-colonization and physical interaction of *P. gingivalis* and other potential pathogens^5^. Given that our findings on mono-species biofilms have demonstrated that among different oral species only *P. gingivalis* can efficiently exploit such a metabolic plasticity to upregulate *fimA* expression, our working hypothesis was that such a metabolic plasticity of *P. gingivalis* may foster biofilm formation of oral species upon co-existence and adherence to *P. gingivalis*, as previously reported^59,60^. To test this hypothesis, we applied a model multispecies system comprised of *P. intermedia*, *T. forsythia*, *S. gordonii*, and *F. alocis*, but in the presence or absence of *P. gingivalis* strain 381; respective biofilms were assessed in the human serum-based medium with and without adding pyruvate. In the absence of *P. gingivalis*, these species were unable to form biofilms in the human serum-based medium, but robust biofilms could develop in the presence of *P. gingivalis* that were significantly augmented upon addition of pyruvate (Fig. 6a). Quantification of the genomic DNA of individual populations using qPCR and 16S ribosomal RNA (rRNA) gene primers showed that the emerging multispecies biofilms mainly consist of *P. gingivalis*, *S. gordonii*, and to a lesser degree, *T. forsythia, P. intermedia*, and *F. alocis*; the constituting populations were also proportionally augmented upon adding pyruvate in the medium, except for *F. alocis* whose abundance was reduced by addition of pyruvate (Fig. 6b). Overall, these findings highlight further the central role of *P. gingivalis* in exploitation of nutritional condition of the subgingival niche to promote the structure and function of subgingival microbial communities.

Furthermore, given that FimA has been implicated as a requirement for colonization and invasion of host cells by *P. gingivalis*^42,61^, we sought to investigate the impact of the availability of pyruvate on *P. gingivalis* capability in colonizing and invading the primary human gingival epithelial (HGEP) cells. First, we confirmed that *P. gingivalis* could form biofilms in RPMI 1640 medium supplemented with 5% human serum, a defined medium used to propagate the primary HGEP cells; still, addition of pyruvate to this medium could increase the biofilm growth of *P. gingivalis* strain 381 by eight times (Supplementary Fig. 8). Then, the interaction of the primary HGEP cells with *P. gingivalis* (multiplicity of infection (MOI) of 100 for 1 h) was performed under both anaerobic and aerobic conditions with and without adding pyruvate. Our data showed that exogenous pyruvate significantly increased the efficacy of the adhesion of *P. gingivalis* to the primary HGEP cells by 2.7 times under anoxia and 2.1 times under normoxia (Fig. 6c). Interestingly, the invasion event was not impacted by adding pyruvate under anoxia, while adding pyruvate under aerobic condition remarkably increased the capability of *P. gingivalis* to invade the primary HGEP cells by 2.7 times (Fig. 6d). Hence, this finding indicated that pyruvate input synergizes with human serum components to promote colonization and invasion of host cells by *P. gingivalis*, while host-mediated mechanisms that are optimally active under normoxia are necessary for accomplishing the invasive event as previously addressed by other studies^46,48,62,63^.

## Discussion

The results presented in this study reveal an underlying metabolic plasticity of *P. gingivalis*, a key player in the etiology of periodontitis. Using defined growth conditions simulating the subgingival niche, we identified a metabolic coupling system in *P. gingivalis* that enables the utilization of pyruvate and lactate. The data show that this metabolic system modulates biofilm-to-migration transition, as well as colonization and invasion mainly through the regulation of fimbrial adhesion; however, further investigations are ongoing to fully reveal its mechanistic basis. Monocarboxylates, like pyruvate and lactate, are ubiquitous carbon and energy sources in the intracellular and extracellular milieus of host cells and GCF; the latter constitutes primary nutritional flow in the microenvironment of subgingival niche^5,26,29,31–35,37–40^, but how such nutritional parameters determines the ecophysiological performance of resident microbiota is not clear. Importantly, the findings presented in this study align with previous reports where various growth conditions and media were used to evaluate the nutritional requirements of oral *Bacteroidetes*, including *P. gingivali*s. Specifically, it was shown that *P. gingivalis* consumes lactate from the culture medium for proliferation under anaerobic conditions^64,65^, while under microaerophilic condition less utilization of lactate was detected^65^. Further, it was shown that cross-feeding of small metabolites, such as lactate, pyruvate, and succinate, between two or more species is an important factor for determining physiological status or growth stimulation of *P. gingivalis*^27,66,67^.

**Fig. 7:**
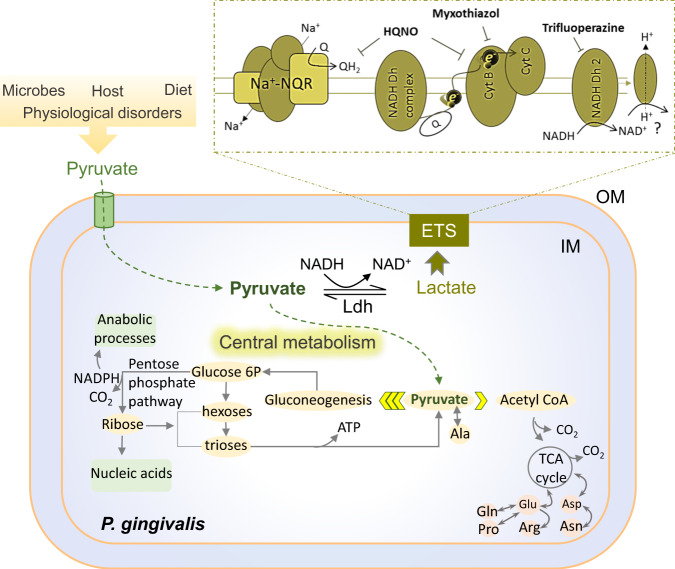
Proposed model of possible bioenergetics system and metabolism of the monocarboxylates pyruvate and lactate in *P*. *gingivalis* that are deduced from the effectiveness of applied uncouplers in RF assay and metabolomic analysis. Pyruvate availability in the intracellular and extracellular milieu of host cells is impacted by various external factors. *P. gingivalis* efficiently transports exogenous pyruvate inside to couple with the metabolism of serum components toward the enhancement of the PPP intermediates that are required for biosynthetic processes, resulting in the increase of cell proliferation and biomass in the biofilm community. ETS electron transport system, Ldh lactate dehydrogenase, Cyt cytochrome, NADH Dh NADH dehydrogenase, Na^+^–NQR the sodium pumping NADH quinone oxidoreductase, Q quinone, e electron, NAD nicotinamide adenine dinucleotide, NADPH nicotinamide adenine dinucleotide phosphate, Glucose 6P glucose-6-phosphate, Glu glutamic acid, Asp aspartic acid, Gln glutamine, Pro proline, Arg arginine, Asn asparagine, Ala alanine, ATP adenosine triphosphate; HQNO 2-heptyl-4-hydroxyquinoline N-oxide, IM inner membrane, OM outer membrane.

Here, our study revealed that it is metabolic coupling that allows *P. gingivalis* to consume exogenous pyruvate as an important source of carbon and energy (Fig. 7), while its combination with amino acids fermentation can maintain the redox and bioenergetic homeostasis of the cells in a nutritionally complex environment of the oral cavity and human cells. Importantly, our findings have demonstrated that the production of FimA fimbria is regulated by the availability of pyruvate and lactate in coordination with the bioenergetic state; hence, redox-changing molecules, such as pyruvate and lactate, can be important input signals in the regulation of fimbria-mediated functional outputs, such as surface attachment, biofilm formation, microbe–microbe interactions, and adherence to host cells. These findings are consistent with our previous study demonstrating that pyruvate availability does not allow *P. gingivalis* to transition to surface translocation under migration-inducing conditions^19^, instead it promotes biofilm growth by enhancing the production of fimbrial adhesions. Despite the fact that different strains of *P. gingivalis* with different biofilm-forming capacity are able to utilize exogenous pyruvate to enhance biofilm formation under our experimental condition, our data indicates that the biofilm-forming capacity of these strains is highly variable.

To date studies have shown that *P. gingivalis* does not utilize carbohydrates as carbon and energy sources^68,69^, our work has revealed that exogenous pyruvate can be an important alternative carbon and energy source that significantly rewires the central metabolism of this slow growing anaerobe toward de novo biosynthesis of phosphorylated and non-phosphorylated carbohydrates via boosting the PPP and gluconeogenesis, a pronounced metabolic shift that apparently is not achievable via the utilization of proteinaceous components alone (Fig. 7). These pathways are important metabolic routes for providing metabolic intermediates, such as ribose-5-phosphate rather than ATP that are required for biosynthesis processes in proliferating cells; as well as regulating “go or grow” cellular programs^57,70–73^; however, it is not clear how this metabolic reprogramming contributes to bacterial pathogenesis. Our research demonstrates that in contrast to the widespread saccharolytic lifestyle, which heavily relies on sugar consumption to modulate the rate of the PPP and gluconeogenesis^55,57^, *P. gingivalis* exploits pyruvate availability to do so. Analogous to the oxidative phase of the PPP that diverts glucose away from glycolysis toward the production of other intermediates^74^, pyruvate input leads to the generation of important PPP-derived phosphorylated carbohydrates, such as ribose-5-phosphate and ribulose-5-phosphate for anabolic processes in *P. gingivalis*; but, exact direction and the flux of pyruvate-derived carbon remain to be fully resolved using isotopic labeling of pyruvate and tracing. These phosphorylated intermediates are necessary for nucleotide biosynthesis in proliferating cells, a process that needs to be tightly coordinated with cellular energy levels determined by the optimal ratio of energy resources, such as [NADH]/[NAD^+^] cofactors that also act in coordination with bioenergetic systems, such as Na^+^-NQR complex^56,57,74,75^. Hence, we showed that pyruvate availability greatly reduces the levels of [NAD^+^] cofactor in the cells, while enhancing the expression of biofilm-formation-related features, such as FimA fimbria whose biogenesis is a highly energy-demanding biosynthetic process. Previously, it was shown that fimbriation is a sensitive process to altered activity of the Na^+^–NQR complex^75–77^, and that our current study showed that HQNO uncoupler significantly downregulates fimbrial expression in *P. gingivalis*; thus, biofilm formation was reduced by bioenergetic uncouplers.

Such metabolic plasticity is a pronounced evolutionary advantage for the survival and persistence of *P. gingivalis* in a highly dynamic and ever-changing environment of the oral cavity. Our data showed that, among various oral species, *P. gingivalis* strains are capable of harnessing nutritional values of serum components, which is necessary for tight coupling with pyruvate metabolism. Such specialization in harvesting diverse nutrients is of paramount importance for the complex metabolic crosstalk within polymicrobial communities. Consistent with the concept that *P. gingivalis* plays a key role during polymicrobial infections^5^, our experimental findings demonstrate that *P. gingivalis* plays an ancillary role in the development of polymicrobial biofilms upon establishing physical and cross-feeding networks with accessory pathogens, such as *S. gordonii, T. forsythia*, and *P. intermedia*, which are also implicated in the etiology of periodontitis^5,60,78,79^. Although the molecular basis of *P. gingivalis* interaction with other species, in particular streptococci, has been frequently addressed in different works^59,60,80–82^, our study has emphasized the use of growth conditions that mimic ecophysiology of subgingival niche and offers a biologically relevant model system for studying in situ interactions between various oral pathogens. Using these experimental parameters, we found that pyruvate availability favors the expression of FimA fimbria in *P. gingivalis* and enhances interspecies interactions and co-colonization of oral pathogens. Moreover, corresponding to the upregulation of FimA fimbria, the adherence of *P. gingivalis* to the host cells is boosted. Importantly, we showed that the invasive event and internalization into the primary HGEP cells was enhanced by adding pyruvate only when oxygen is available to maintain host cell homeostasis. This is consistent with previous reports demonstrating that following *P. gingivalis* adherence to host cells, and the subsequent invasion process requires host-mediated mechanisms, such as rearrangement of cellular dynamin, actin fibers, and microtubules^46,48,62,63^. In summary, it is evident that *P. gingivalis* harnesses and responds to the entire complement of in-host substrates and metabolites to modulate lifestyle, virulence, and its interaction with the existing microbiota and host cells. The data provide further support for studying dysbiosis in the oral microbiome, which is considered as an important risk factor for the onset of chronic inflammation and a variety of systemic diseases^83–85^.

## Methods

### Bacterial strains, cell culture, growth conditions, and chemicals

*P. gingivalis* strains 381, ATCC 33277 and W50, *C. matruchotii* ATCC14266, and *S. gordonii* DL-1, *S. intermedius* strain F0413, *P. intermedia* strain 17, *F. alocis* ATCC 35896 and *T. forsythia* ATCC 43037, and *F. nucleatum* ATCC 10953 were applied in this study and cultivated from frozen stocks. *P. gingivalis* ∆*sprA*, *P. gingivalis* ∆*sprA* + pTCOW::*sprA*, and *P. gingivalis* + pTCOW were adopted from our previous work^19^. TSBHK containing Trypticase Soy Broth (Becton, Dickinson and Company, Franklin Lakes, NJ, USA), 5 μg/ml hemin, and 1 μg/ml menadione was used for the cultivation of Bacteroidetes species, and N-acetylmuramic acid (10 μg/ml) was added to the medium for the cultivation of *T. forsythia*. For solid culture cultivations, BAPHK [referring to TSBHK supplemented with 5% defibrinated sheep blood (Northeast Laboratory Services, Winslow, ME, USA)] or BHI (BBL™ Brain Heart Infusion Broth; BD Biosciences) were applied. Desired concentrations of agar were made using Bacto™ Agar or Agarose (Low-EEO/Multi-Purpose/Molecular Biology Grade, Fisher BioReagents). For BSA-, HSA-, or human serum-based media, HyClone™ BSA (GE Healthcare Life Sciences), HSA (Sigma; cat# A9511), and CELLect® Human Serum (ICN Biomedicals, Inc.; cat# 2930149) were applied, respectively, in the BS buffer containing 14 mM Na_2_HPO_4_, 10 mM KCl, and 10 mM MgCl_2_, pH 7.3, and 5 μg/ml hemin and 1 μg/ml menadione was added to these media in all experiments. Incubation of all bacterial species was performed at 37 °C in an anaerobic chamber (Coy Lab Products, Grass Lake, MI, USA) with an atmosphere containing 5% hydrogen, 10% carbon dioxide, and 85% nitrogen. The primary HGEP cells (Celprogen) were cultured either in Human Oral Epithelial Cell Culture Complete Growth Media with Serum (Celprogen; cat# M36063-01S) or in RPMI 1640 (Gibco; cat# 11835-030) supplemented with 5% human serum (v/v) at 37°C in 5% CO_2_. Corning^®^ 24-well microplates (CLS3527), pre-coated with poly-L-lysine solution (Sigma, cat# P4832), were used for HGEP cultivation. For performing assays under microaerophilic condition, BD GasPak™ EZ container system and Thermo Scientific™ AnaeroPouch™-MicroAero Gas Generator were applied. All chemicals, reagents, and chemical uncouplers were purchased from Sigma-Aldrich unless otherwise mentioned.

### Biofilm and surface translocation assays

The biofilm and surface translocation assays were performed at 37 °C, inside an anaerobic chamber (COY Lab Products), respectively, using our standard 96-well biofilm protocol and our designed motility chamber, as previously described^19^. For biofilm assay, an overnight culture was washed with pre-reduced BS buffer and pelleted inside anaerobic chamber. Bacterial cells were resuspended in the working medium supplemented with 5 μg/ml hemin and 1 μg/ml menadione to reach the optical density at 600 nm (OD_600_) = 0.25 (5 × 10^9^ cells/ml). A total of 200 μl of the cells was applied in each well and anaerobically incubated for 48 h. The plates containing bacterial biofilms were washed twice by immersing in distilled water to remove free cells, and air dried. A total of 200 μl of 0.1% safranin in water was used to stain each well for 30 min. The plates were washed by immersing twice in distilled water, and air dried. The extraction of the stain from biofilms was performed using 200 μl of 90% ethanol containing 1% sodium dodecyl sulfate for 30 min followed by reading the absorbance at 492 nm. For multispecies biofilm assays, an overnight culture of each species was harvested, washed, and prepared in the working medium as explained above. A total of 40 μl of each species (at OD_600_ = 0.25) was added to each well in a 96-well plate to the final volume of 200 μl (~2 × 10^9^ cells/ml); for those conditions without *P. gingivalis*, the only medium was applied. Incubation was performed anaerobically for 48 h. Biofilms were subjected to safranin staining, as explained above or genomic DNA (gDNA) isolation, as described later.

Surface translocation assay was performed using the motility chamber slide, as described previously^19^. Briefly, a motility chamber slide was made by compressing several layers of sterilized parafilm M (Bemis) on a glass slide followed by fixing of the parafilm with slight heat. The sides of the slide were then sealed with nail polish. The center of the parafilm layer was removed with a sterile scalpel to create a chamber (0.5 cm *H* × 1.5 cm *W* × 3.0 cm *L*). This chamber was then filled with soft agar medium, i.e., BHI medium containing 0.3% agar. The medium was allowed to solidify, and a coverslip inoculated with a tiny dot of cells at the center was inverted and placed on chamber filled with medium and mounted with nail polish. The slide was anaerobically incubated for more than a week. Imaging was performed using an inverted Nikon Eclipse Ti microscope system (Nikon, Tokyo, Japan) inside an anaerobic chamber^19^.

### Biofilm analysis by confocal laser scanning microscopy

Sample preparation was performed as described previously^86^ with some modifications. Inoculum was prepared as described above for biofilm assay. Cells were diluted to OD_600_ = 0.2 and 1 ml of the cells was added to each well of a 12‐well microtiter plate (Greiner Bio‐one) containing a circular glass coverslip. Plates were incubated for 24 h in the anaerobic chamber. Then glass coverslips were gently washed with distilled water to remove free cells and stained with the Invitrogen Live/Dead BacLight Bacterial Viability Kit (Thermo Fisher Scientific). For staining each coverslip, 1 µl of SYTO 9 and 1 µl of propidium iodide (PI) were mixed and added to 1 ml of distilled water. The coverslips were treated with the staining solution for 15 min, followed by washing with 1 ml of distilled water. The coverslips were then removed, placed face down on a glass microscope slide, and biofilms were visualized by a laser scanning confocal microscopy on a Leica SP8 confocal microscope (Leica Microsystems Inc.), using 507/545 nm (SYTO 9) and 614/745 nm (PI) lasers, and ×20 objective. Images were analyzed using IMARIS image analysis software (Bitplane AG).

### Resazurin flourometric assay

This assay was performed in 96-well microtiter flat-bottom plates made of polystyrene (Falcon 353936). Upon optimization procedure, 2 µl of 50 mg/ml resazurin (filter sterilized) in 200 µl of cell suspension with OD_600_ = 0.15 were found ideal for monitoring redox state over 6 h. Cells at the exponential phase in TSBHK medium were cooled down on ice and pelleted at 4700 r.p.m. for 15 min. Cells were washed once with pre-reduced BS buffer and resuspended and diluted (OD_600_: 0.15) in BS buffer containing 5 µg/ml hemin and 1 µg/ml menadione, as working cell suspension. Substrates were prepared as a 25% stock solution in BS buffer (pH 7.3). Redox state was measured fluorometrically (excitation: 554 nm, emission: 593 nm) on the Synergy H1 Hybrid Microplate Reader (BioTek). This experiment was conducted using at least three repetitions for each treatment.

### [NADH]/[NAD^+^] ratio measurements

Cells at the exponential phase (OD_600_:0.5) in TSBHK medium were cooled down on ice and pelleted at 4700 r.p.m. for 15 min. Cells were washed once with pre-reduced BS buffer and resuspended in the same volume of BS buffer containing 5 µg/ml hemin and 1 µg/ml menadione to retain OD_600_ at 0.5. Cell suspensions (4 ml) were supplemented with desired substrates and incubated anaerobically at 37 °C for 6 h (cell doubling time is 3 h). Then, cells were placed on ice, pelleted, and subjected to [NAD^+^] and [NADH] extraction and quantification, using the EnzyChrom NAD^+^/NADH assay kit (BioAssay Systems). The protocol of this kit was followed for separate extraction of NAD^+^ and NADH, and 100 µl of each extraction buffer and 20 µl of assay buffer were applied for 8.7 mg of cells. The standard curve was prepared according to the manufacturer’s protocol using NAD^+^ as standard. The intensity of generated colors was measured on the Synergy H1 Hybrid Microplate Reader (BioTek) at 565 nm.

### cDNA preparation, quantitative reverse transcription PCR, and quantification of individual population within multispecies biofilms

Biofilm assays were set up in parallel with sample preparation for the RNA experiments for monitoring the consistency between the findings. For relative quantification of desired genes, RNA extraction, cDNA preparation, and RT-qPCR assay, we followed the procedures described in our previous work^19^. RNA extraction was performed in the anaerobic chamber to avoid aerobic stress using Direct-zol^™^ RNA MiniPrep Kit (Zymo Research) with slight modification. Briefly, RNA extraction was performed for biofilm cells after removing free and planktonic cells from 96-well plates. Biofilm cells were lysed using 200 µl of TRI Reagent^®^ (Zymo Research) for each well. Debris were removed from collected samples by centrifugation at 14,000 × *g* for 10 min and equal volume of 100% ethanol was mixed with each sample. Each mixture was loaded into a Zymo-Spin^™^ IIC column and centrifuged at 14,000 × *g* for 30 s followed by washing using 400 µl of RNA wash buffer. DNA residues were completely digested and removed from columns in two rounds, each by adding 80 µl of DNase I and DNA Digestion Buffer (see Direct-zol^™^ RNA MiniPrep Kit protocol), and incubating at 30 °C for 1 h followed by centrifugation at 14,000 × *g* for 30 s. Second round was followed by washing columns using 400 µl of Direct-zol^™^ RNA PreWash and 700 µl of RNA Wash buffers. Pure RNA crude was eluted twice using 15 µl of DNase/RNase-free water. Quality control of RNA samples was performed using Qubit^®^ 2.0 Fluorometer (ThermoFisher/Invitrogen, Grand Island, NY). For RT-qPCR, the reverse transcription reactions were performed by using the RNA to cDNA EcoDry Premix (Random Hexamers) kit (Clontech Laboratories, Inc.). Briefly, for each sample, 2500 ng of isolated RNA was added into 20 µl of reaction solution as final volume. Reaction was performed at 42 °C for 60 min using a Bio-Rad T100^TM^ thermocycler followed by stopping it at 70 °C for 10 min. For relative quantification of desired genes, qPCR assays were conducted in a 20 µl total volume containing 1 µl of 1:10 diluted cDNA, 0.5 µM of each primer (Supplemental Table 2), 6 µl of PCR grade water, and 10 µl of 2× iQ SYBR Green Supermix. Amplification and detection of product were performed using the CFX96 Touch™ Real-Time PCR Detection System (Bio-Rad) and cycling condition was applied as the following: 95 °C for 3 min, and then 39 cycles of 95 °C for 20 s, 55 °C for 20 s, and 72 °C for 20 s and fluorescence was detected after each cycle. In each experiment, the target and control samples were amplified in the same plate and conducted in triplicate and normalized internally using simultaneously the average cycle quantification (Cq) of the reference gene. To confirm specificity of the amplified products, automated melting curve analysis was performed.

Supplementary Table 4 represents the primers used in RT-qPCR for quantification of relative gene expression levels. For quantification of the individual population within multispecies biofilms using qPCR, we applied the standard curve method described previously^87,88^ with slight modification. Briefly, the genomic DNA of each species was isolated from 1 ml cultures using The Wizard^®^ Genomic DNA Purification Kit (Promega). Using serial dilutions of gDNA (10–0.001 ng DNA) and 16S rRNA gene primers (Supplementary Table 5) in the qPCR assay (95 °C for 3 min, and then 39 cycles of 95 °C for 20 s, 55 °C for 20 s, and 72 °C for 20 s)^19^, the standard curve of each species was constructed by plotting the logarithm of the average Cq value of reference against a defined concentration of gDNA for each species. For the quantification of bacteria in multispecies biofilm samples, multispecies biofilms were prepared as explained above and gDNA samples were isolated. The concentration of the extracted gDNA mixture was determined and a 10 ng of mixed gDNA and species-specific primer pairs (10 μM for each primer) was used in a total reaction volume of 20 μl for qPCR analysis, as described^19^. From the obtained Cq values, absolute DNA concentration was calculated for each species by plotting on the linear regression, and the relative abundance was calculated from the total gDNA that was obtained in each biofilm biomass.

### Global metabolomic analysis on the metabolome of biofilm cells

Preparation of biofilm cells for metabolomic analysis was similar to the procedure explained in the biofilm preparation section. HSA (0.4%) and human serum (5%) in the BS buffer, with or without pyruvate addition, were used as the working media. After 48-h incubation of 96-well plates, the medium was removed from wells. Biofilm cells were washed with cold phosphate-buffered saline (or PBS, pH 7.2), collected by pipetting, and transferred into tubes. After a centrifugation at 4700 × *g* for 30 min at 4 °C, collected samples were freeze-dried, weighed, and subjected to further purification using the solvent system consisting of acetonitrile:methanol:acetone (8:1:1)^19^. For global metabolomic analysis, extracts were pre-normalized to the sample protein content of 500 µg/ml prior to LC–MS/MS analysis^19^. LC–MS/MS analysis was performed on a Thermo Q-Exactive Oribtrap mass spectrometer with Dionex UHPLC and autosampler. All samples were analyzed in positive and negative heated electrospray ionization with a mass resolution of 35,000 at *m*/*z* 200 as separate injections. Separation was achieved on an ACE 18-pfp 100 × 2.1 mm, 2 μm column with mobile phase A as 0.1% formic acid in water and mobile phase B as acetonitrile. The flow rate was 350 μl/min with a column temperature of 25 °C. A 4 and 2 μl samples were injected for negative and positive ions, respectively. Data from positive and negative ion modes were separately subjected to statistical analyses, and all subsequent data analyses were normalized to the sum of metabolites for each sample. The open-source software MZmine^89^ was used to identify features and deisotopes, and the MetaboAnalyst software^90^ was applied for comparative and statistical analyses.

### Adhesion and invasion assay

This assay was performed using a method described by Inaba et al.^91^. To this end, we used *P. gingivalis* strain 381 and primary HGEP cells growing in RPMI 1640 medium (without antibiotic) supplemented with 5% human serum. First, we confirmed that *P. gingivalis* can form biofilms in RPMI 1640 medium supplemented with 5% serum as explained in the biofilm preparation section. For invasion assay, an overnight culture of *P. gingivalis* strain 381 cells grown in 2 ml of TSBHK was harvested and washed with RPMI 1640 medium. The bacterial pellet was resuspended in 2 ml of RPMI 1640 medium that was used as the stock bacterial culture (colony-forming unit (CFU) = 1.5 × 10^7^). On the other hand, primary HGEP cells were grown to 90% confluence in RPMI 1640 medium supplemented with 5% serum in 24-well microplates (pre-coated with 0.01% poly-L-Lysine solution). The primary HGEP cells in each well were washed with 1 ml of RPMI 1640 medium. Two sets of plates containing the primary HGEP cells were used to infect with *P. gingivalis* cells from the stock bacterial culture at a MOI of 100. In each set, a group of wells (*n* = 4) were supplemented with pyruvate to the final concentration of 0.5%. After adding the bacterial cells to each well, a set of these plates was aerobically incubated, while another set was incubated in an anaerobic chamber; both at 37 °C for 1 h. After incubation, all plates were washed three times with pre-reduced PBS buffer (1 ml per well) under anaerobic condition. For total adhesion and invasion assessment, cells were lysed with 1 ml of sterile distilled water for 15 min, and serial dilutions of the lysates were plated on BAPHK and were anaerobically incubated for CFU measurement. For invasion assessment, first, extracellular bacteria were killed using metronidazole (200 μg/ml) and gentamicin (300 μg/ml) for 1 h, then infected cells were lysed for CFU enumeration. The results were calculated from recovered CFU values as a percentage of total bacteria that were applied as the initial inocula.

### Statistical analysis

Statistical analysis for metabolomics data was based on univariate analysis by ANOVA that was performed separately on the positive and negative ion data from all data sets. For other data, the Shapiro–Wilk test was used to evaluate the normality of the distribution of the data. It was indicated that the data were normally distributed (Shapiro–Wilk test: *p* > 0.05); therefore, the results were statistically analyzed by ANOVA followed by post hoc Tukey’s honestly significant difference (HSD) test for pairwise comparisons, using XLSTAT statistical add-in software for Microsoft Excel 14.0. GraphPad Prism version 8.0.1 was applied for performing Student’s *t* test in pairwise comparisons. All experiments were conducted using at least three repetitions. Differences in the data were considered significant when the probability value was <5.0% (*p* value <0.05).

### Reporting summary

Further information on experimental and research design is available in the Nature Research Reporting Summary linked to this article.

## Supplementary information

Supplementary Information

Reporting Summary

## Data Availability

The authors declare that the data supporting the findings of this study are available within the paper, and its supplementary information files or from the corresponding author upon request.
