# Supplementary material for: Metabolic plasticity enables lifestyle transitions of Porphyromonas gingivalis
Source: NPJ Biofilms Microbiomes. 2021 May 24;7:46. doi: 10.1038/s41522-021-00217-4 (PMC8144566; doi:10.1038/s41522-021-00217-4)
Supplement: Supplementary file 1 — Supplementary Information [file 41522_2021_217_MOESM1_ESM.pdf]

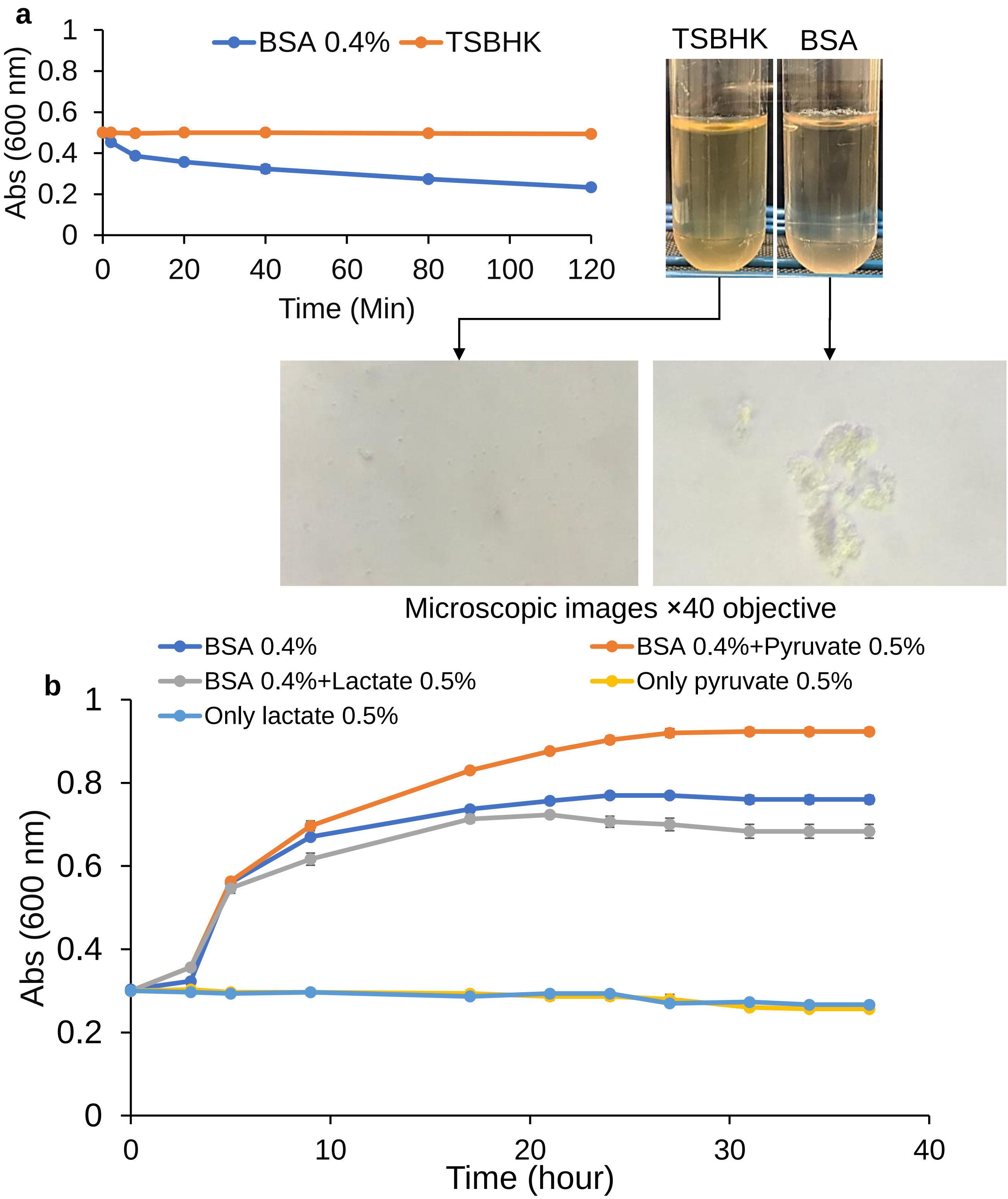

Supplementary Figure 1 (A) Optical density measurements (left) show time-dependent settling of cells grown in BSA but not in TSBHK. The medium is turbid in TSBHK cultures but is translucent in BSA cultures (right). (B) Optical density assay (OD_{600}) shows the effect of BSA and the monocarboxylates pyruvate and lactate on the proliferation of *P. gingivalis*. *P. gingivalis* effectively utilizes BSA for proliferation while it cannot utilize pyruvate and lactate as sole carbon and energy sources. However, in the presence of BSA, pyruvate and lactate are incorporated into the metabolism of serum components; pyruvate significantly enhances proliferation and lactate has an inhibitive effect. Graphs are created upon mean OD_{600} ± standard error (n = 3).

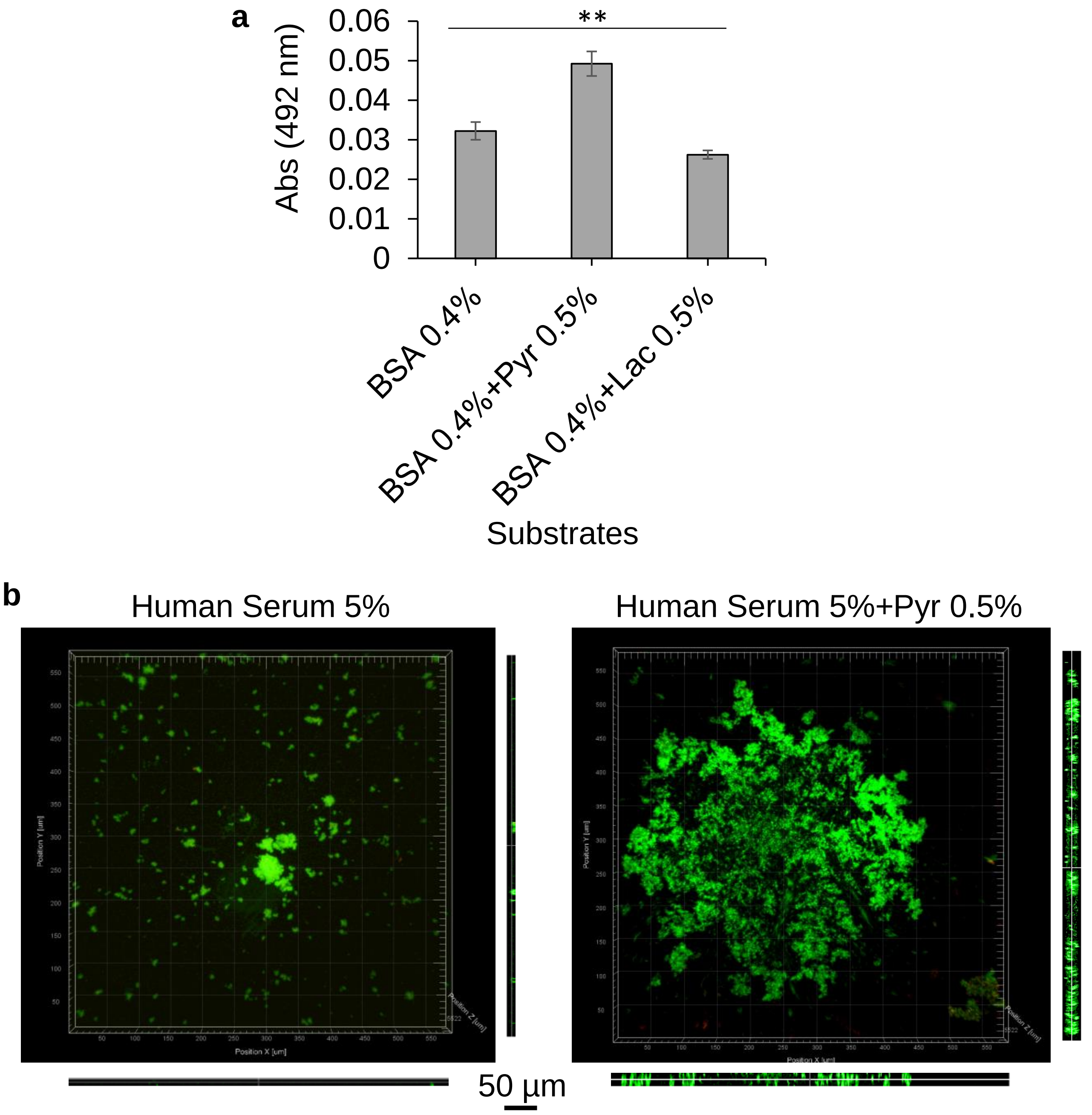

Supplementary Figure 2 (A) Biofilm assay under microaerophilic condition shows that pyruvate significantly enhances *P. gingivalis* biofilm formation and lactate has an inhibitive effect. (B) Biofilm architecture of mutants producing acetylated and nonacetylated alginates. Images of 24-hour biofilms were acquired by CLSM. Top and side views show that pyruvate significantly promotes surface attachment and biofilm development by *P. gingivalis*.

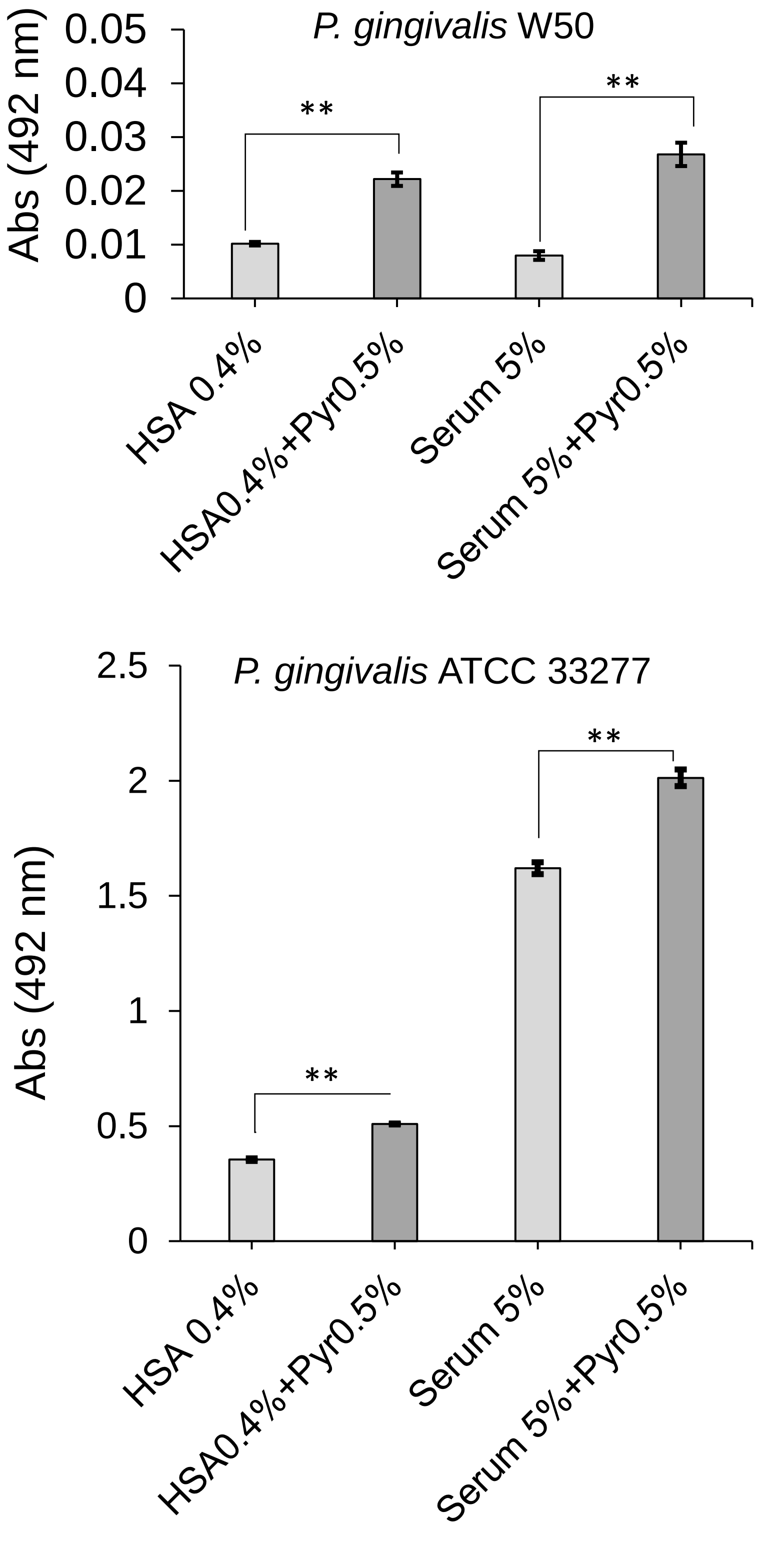

Supplementary Figure 3 Supplementation of HSA or human serum with pyruvate enhances biofilm formation of *P. gingivalis* W50 (poor biofilm former) and ATCC 33277 (weak biofilm former). Graph represents mean biomass of biofilms ± SE (n = 5) at 48 h, as determined by safranin staining. Biofilm biomasses were normally distributed (Shapiro-Wilk test: P > 0.05), and thus analyzed using ANOVA and asterisks indicate pairs of significantly different values (*post hoc* Tukey's HSD test: **, $p < 0.01$).

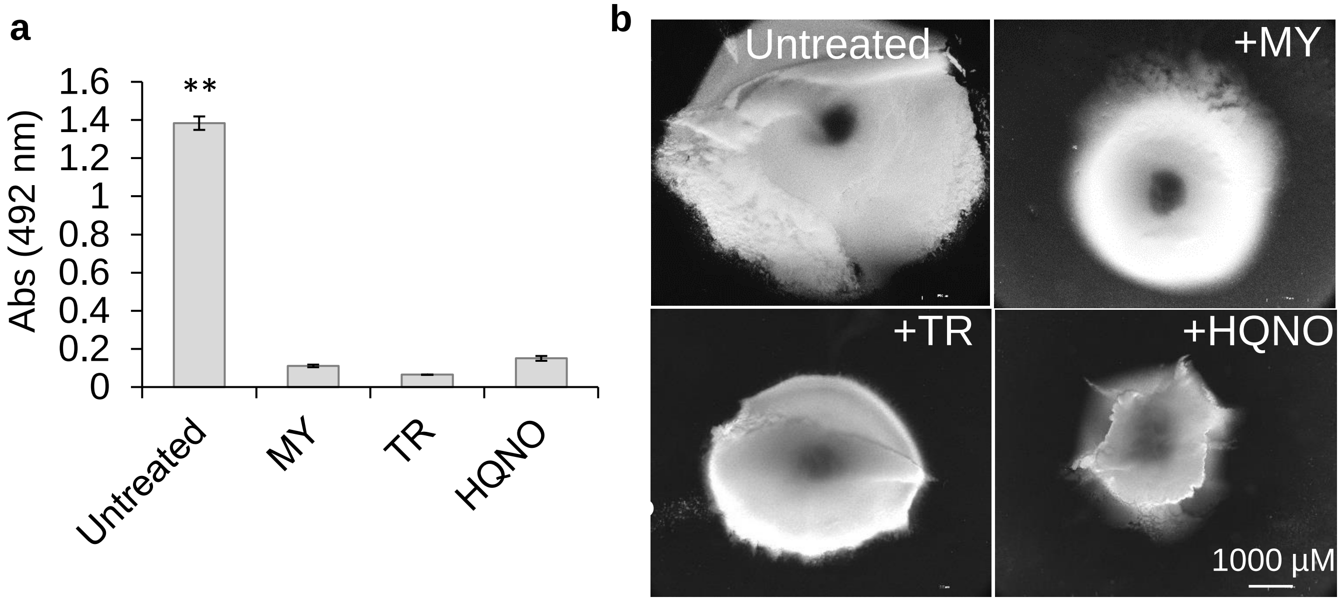

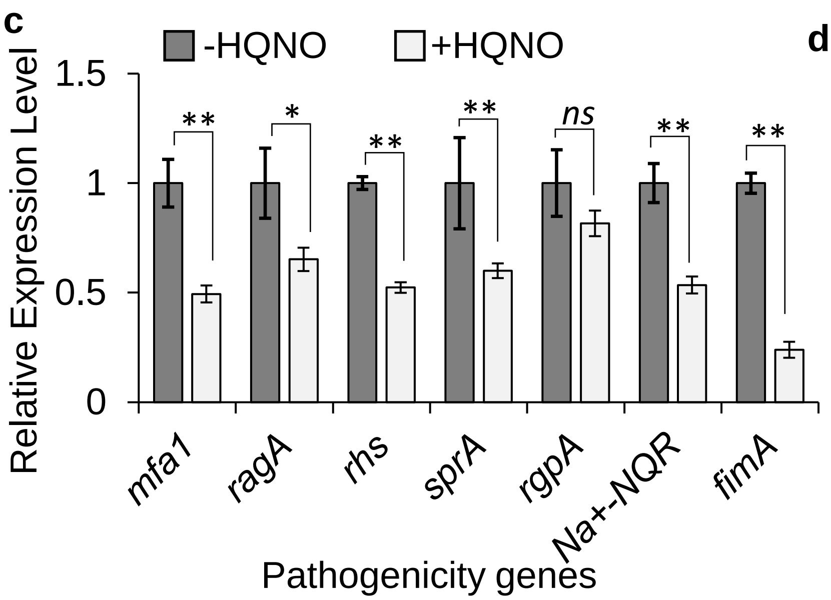

d

Gene	HQNO
ragA	-
rhs	-
sprA	-
rgpA	N
nqr	-
mfa1	-
fimA	- -

No change (N); (+) Upregulation; (-) Downregulation

Supplementary Figure 4 The chemical uncouplers myxothiazol (MY), triflouperazin (TR), and 2-heptyl-4-hydroxyquinoline *N*-oxide (HQNO) (10 µg/ml) are inhibitive to biofilm formation (A) and surface translocation (B). These assays were performed in BHI medium because it provides an optimal condition for promoting *P. gingivalis* surface translocation. (C) RT-qPCR analysis and the relative quantification of gene expression show that that despite the fact that HQNO (10 µg/ml) reduces the expression levels of *ragA*, *rhs*, *mfa1*, *sprA*, and *nqr* genes by up to two times, *fimA* expression is downregulated by almost 5 times, indicating the expression of fimbrial adhesions is highly regulated in coordination with the bioenergetic status of the cells. (D) Table summarizes RT-qPCR results and shows the correlation of HQNO addition and the expression of pathogenicity genes. The data in graphs represent the means ± SD error for at least four replicates, and asterisks indicate pairs of significantly different values (*post hoc* Tukey's HSD test: *, $P < 0.05$; **, $P < 0.01$; *ns*, not significant). Abs: absorbance; HQNO: 2-heptyl-4-hydroxyquinoline N-oxide; TR: trifluoperazine; MY: myxothiazol.

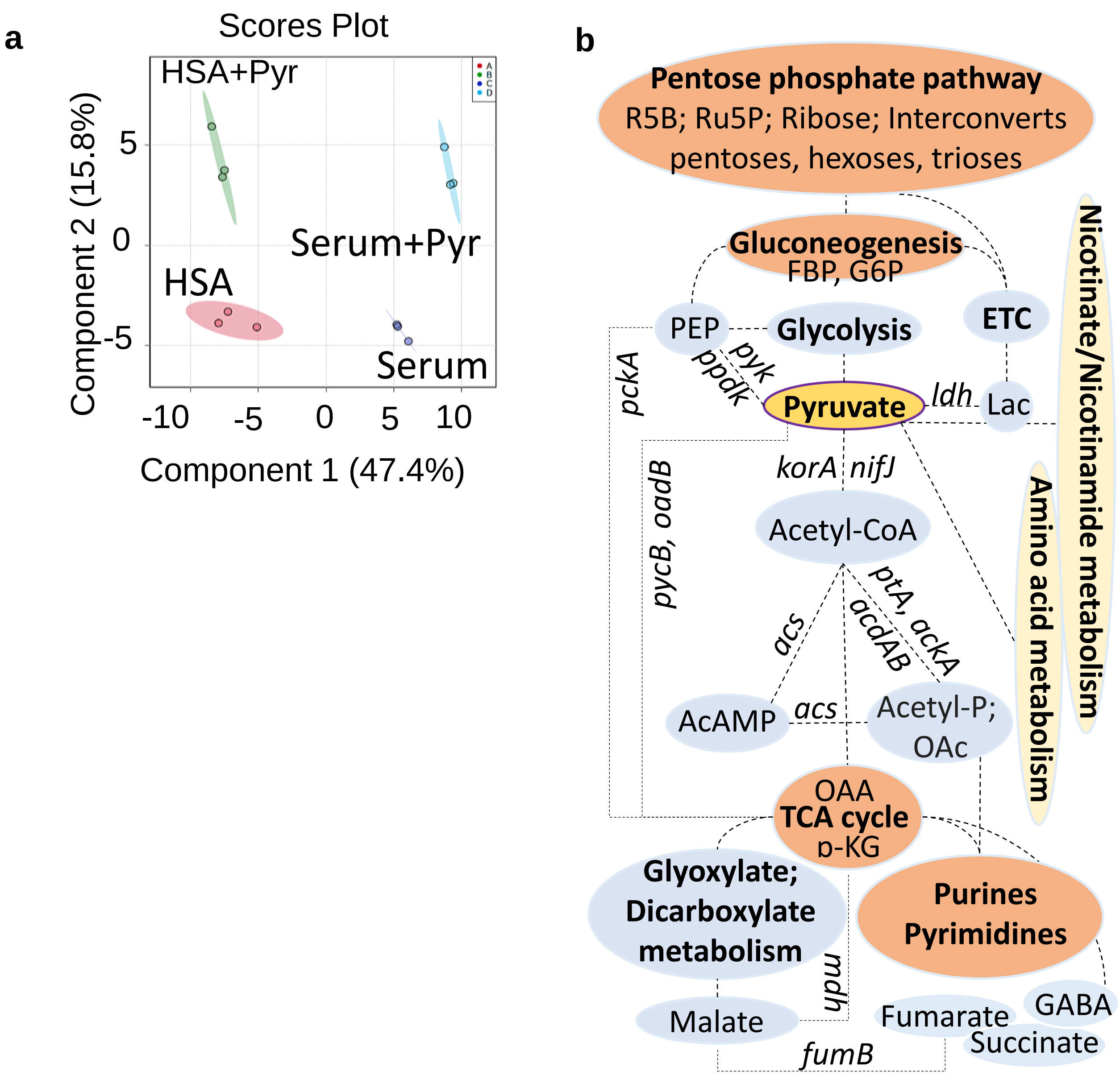

Supplementary Figure 5 (A) Scores plot of principal component analysis (PCA) of a multi-group comparison shows metabolite population distribution among all given growth conditions. (B) Canonical metabolic networks associated with pyruvate metabolism in *P. gingivalis* that were predicted and visualized using MetScape visualization tool. Italic letters represent genes that are predicted in *P. gingivalis*.

a

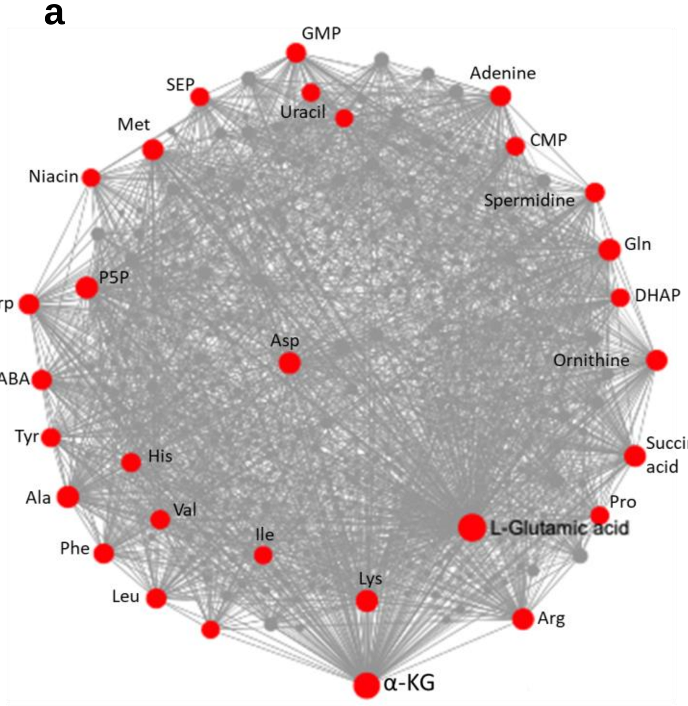

Supplementary Figure 6 (A) Identified metabolites with *p*-value < 0.05 are mapped onto the interaction network. The size of the circles correspond to the node degree (the number of links a node has to other nodes) and betweenness centrality (the degree of centrality a node has in a network) and metabolites with high values of these parameters play as metabolic hubs in the cells. (B) Box-plots showing distribution of average total metabolites (*t* test, *p*-value < 0.05) in the metabolome of biofilm cells upon concomitant utilization of HSA and pyruvate when compared with HSA or human serum-only medium.

b

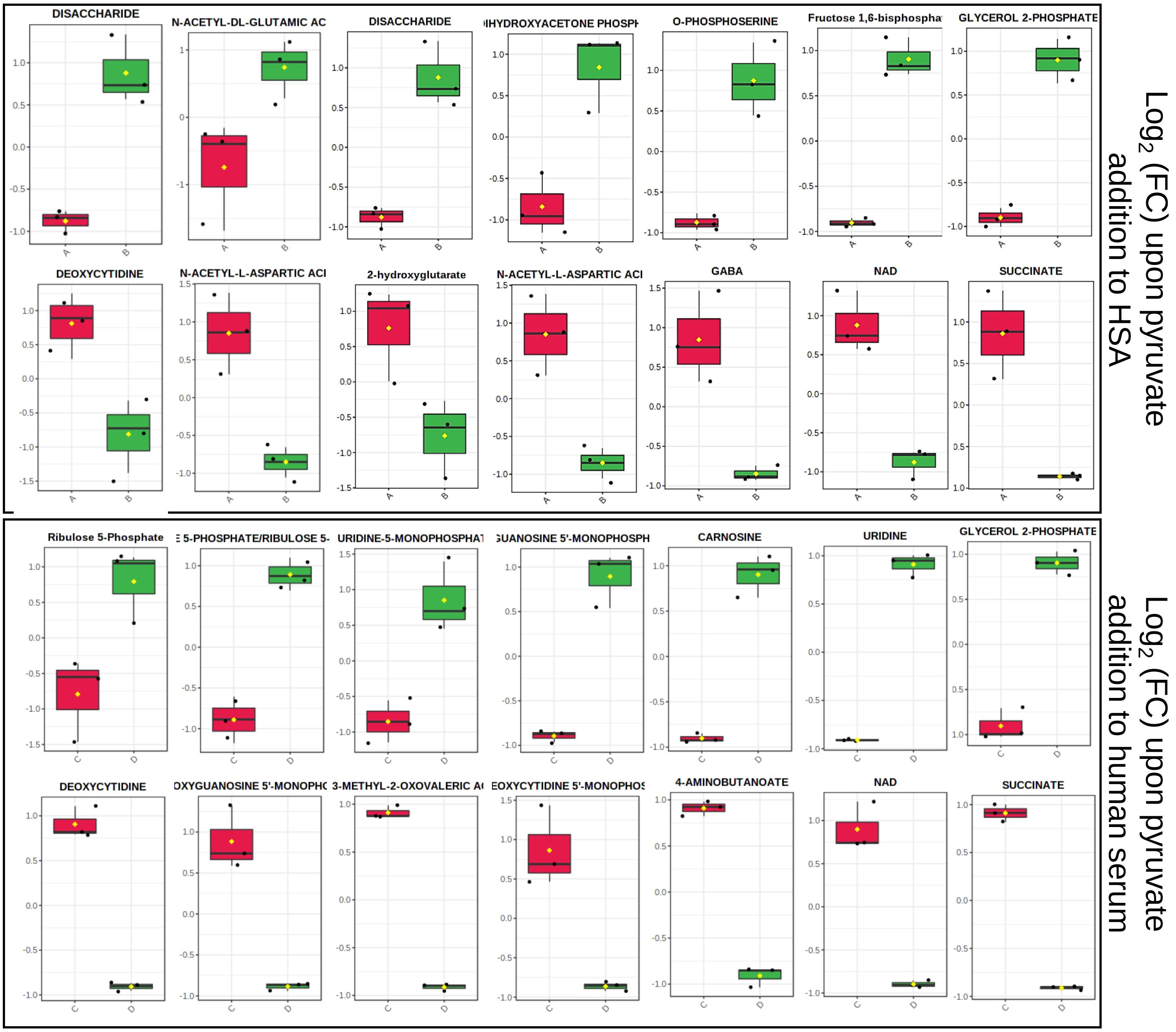

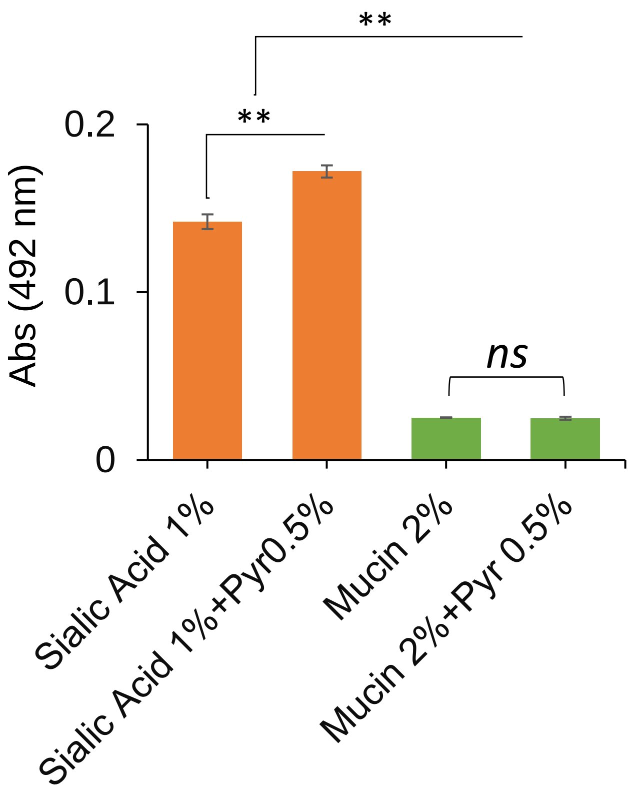

Supplementary Figure 7 Biofilm assay shows *P. gingivalis* can utilize free n-acetylneuraminate as sole nitrogen/carbon source, but not mucin-associated n-acetylneuraminate, to form biofilm that is augmented when pyruvate is added. Graph represents mean biomass of biofilms ± SE (n = 5) at 48 h, as determined by safranin staining. Biofilm biomasses were normally distributed (Shapiro-Wilk test: P > 0.05), and thus analyzed using ANOVA test. Asterisks indicate pairs of significantly different values (*post hoc* Tukey's HSD test: **, $p < 0.01$; *ns*, not significant).

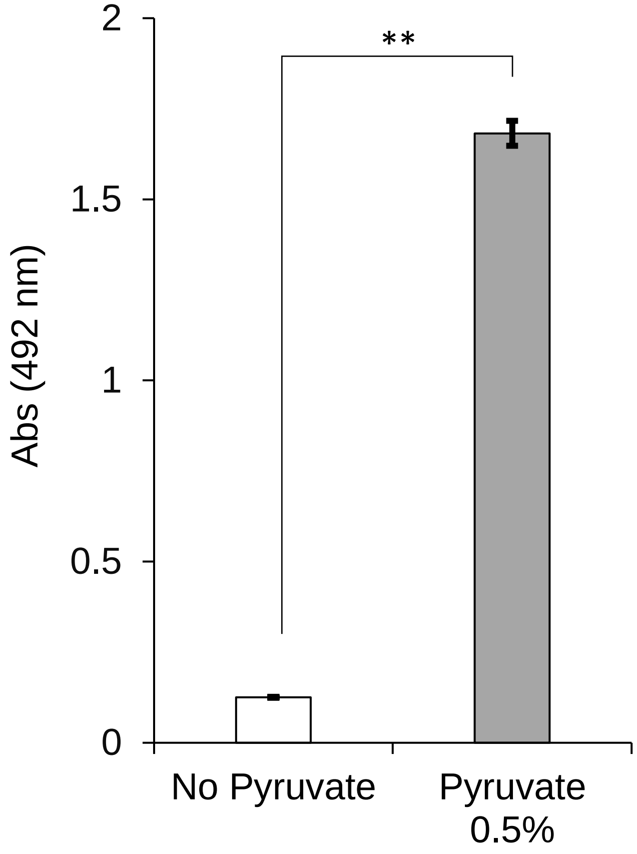

Supplementary Figure 8 Biofilm assay shows that *P. gingivalis* forms biofilm in RPMI 1640 medium supplemented with 5% human serum; biofilm was augmented upon pyruvate addition. Graph represents mean biomass of biofilms ± SE (n = 5) at 48 h, as determined by safranin staining. Biofilm biomasses were normally distributed (Shapiro-Wilk test: $P > 0.05$), and thus analyzed using ANOVA test. Asterisks indicate pairs of significantly different values (*post hoc* Tukey's HSD test: **, $p < 0.01$).

Supplementary Figure 9 Box plots of all experiments displaying the data distribution through their quartiles.

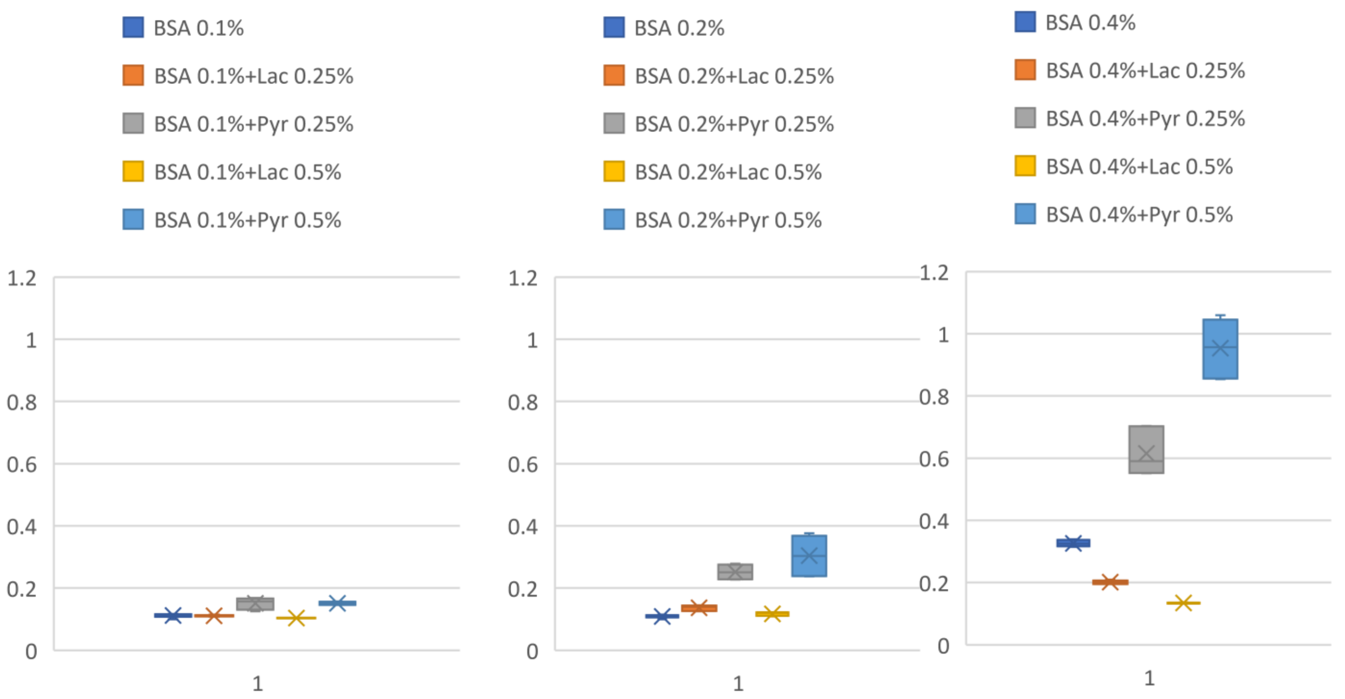

Box plot of Fig. 1A displaying the data distribution through their quartiles

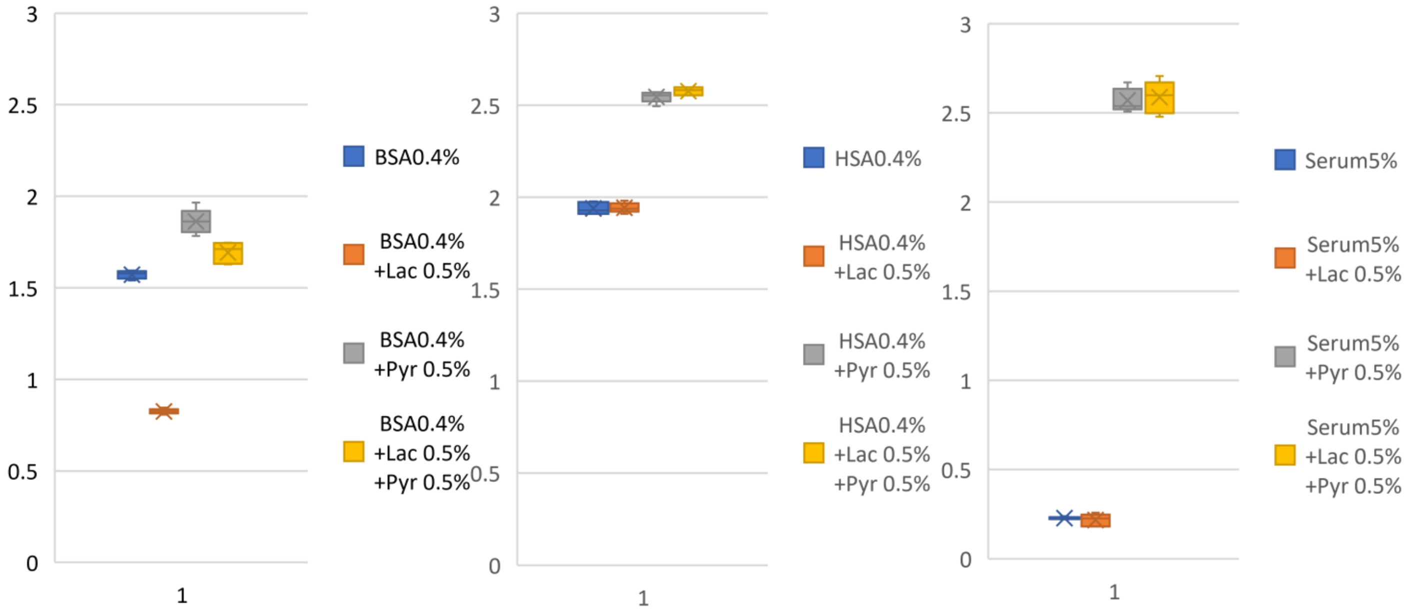

Box plot of Fig. 1B displaying the data distribution through their quartiles

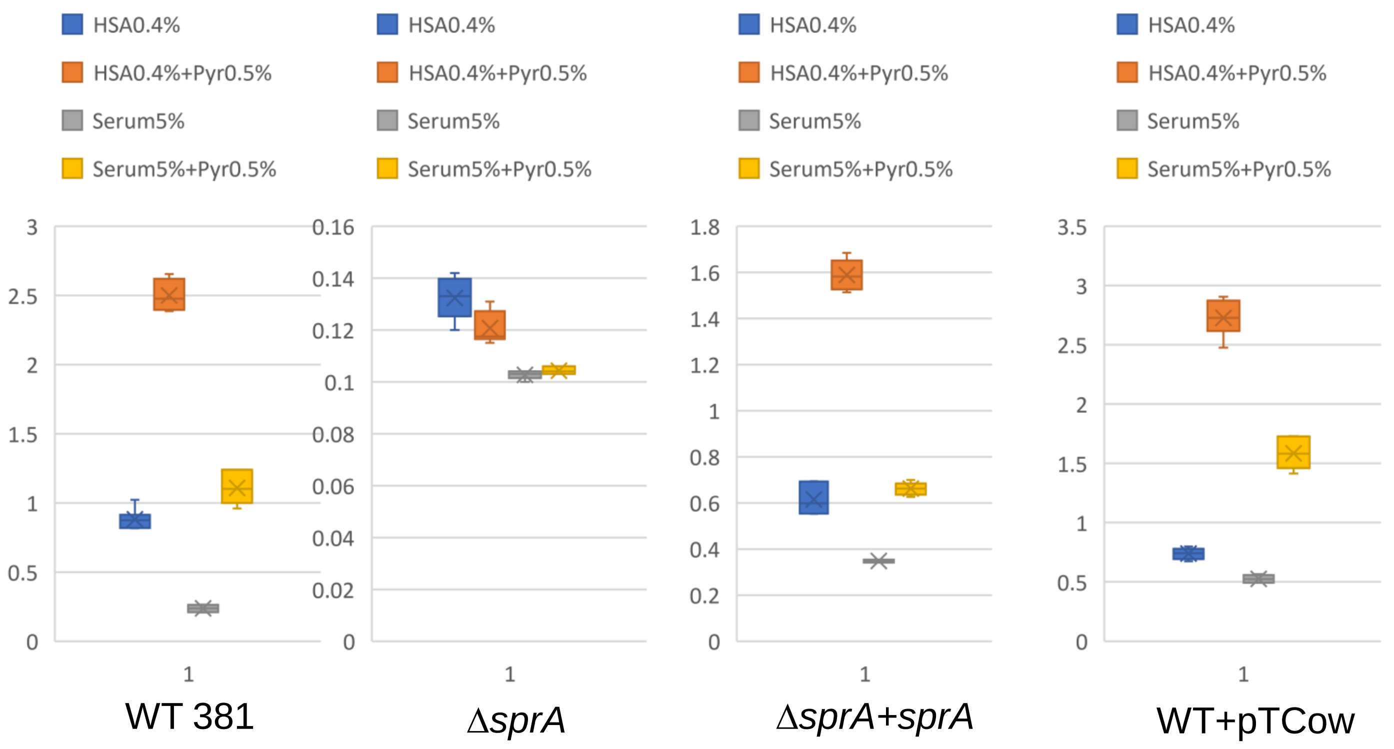

Box plot of Fig. 2A displaying the data distribution through their quartiles

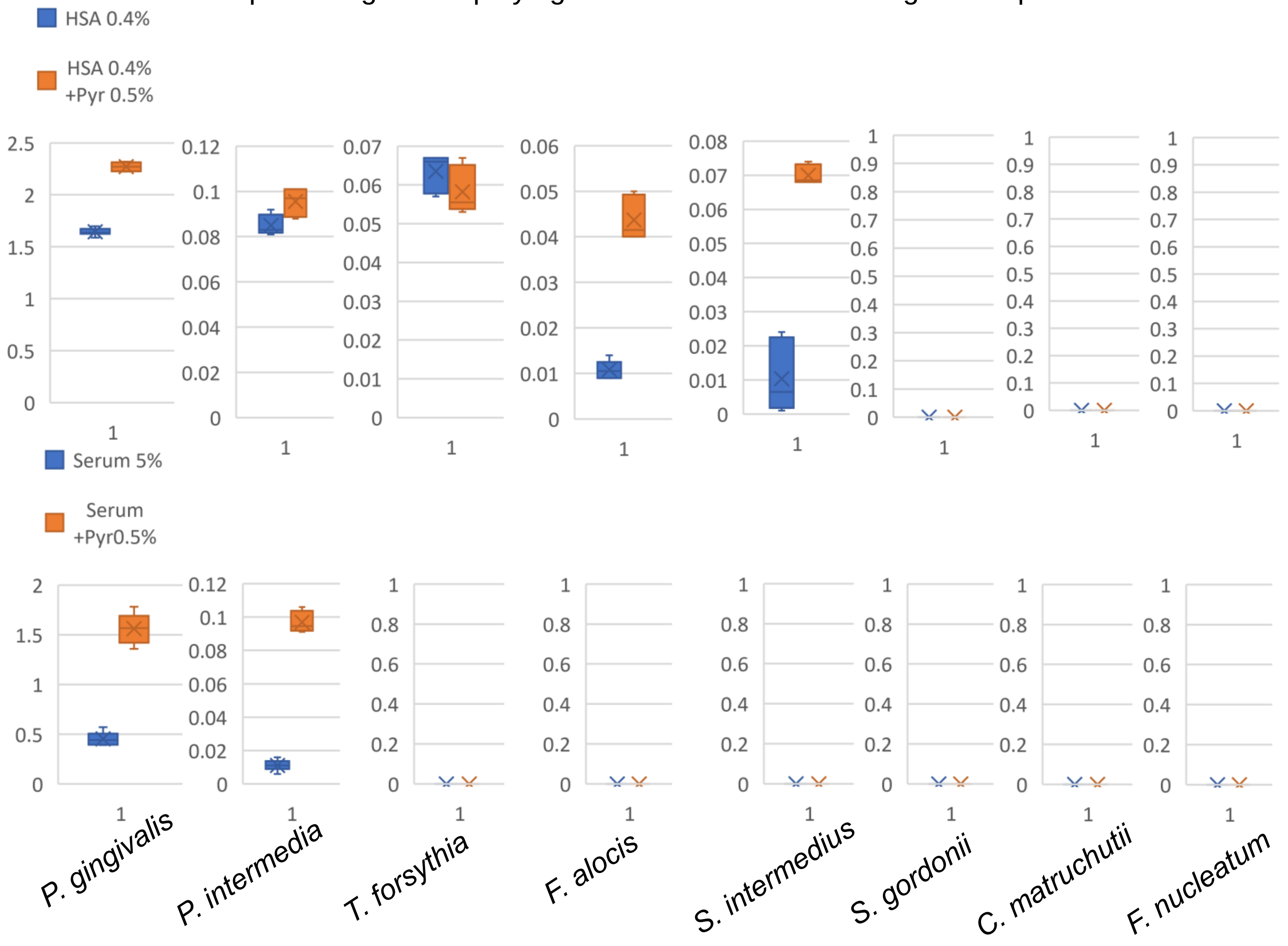

Box plot of Fig. 2B displaying the data distribution through their quartiles

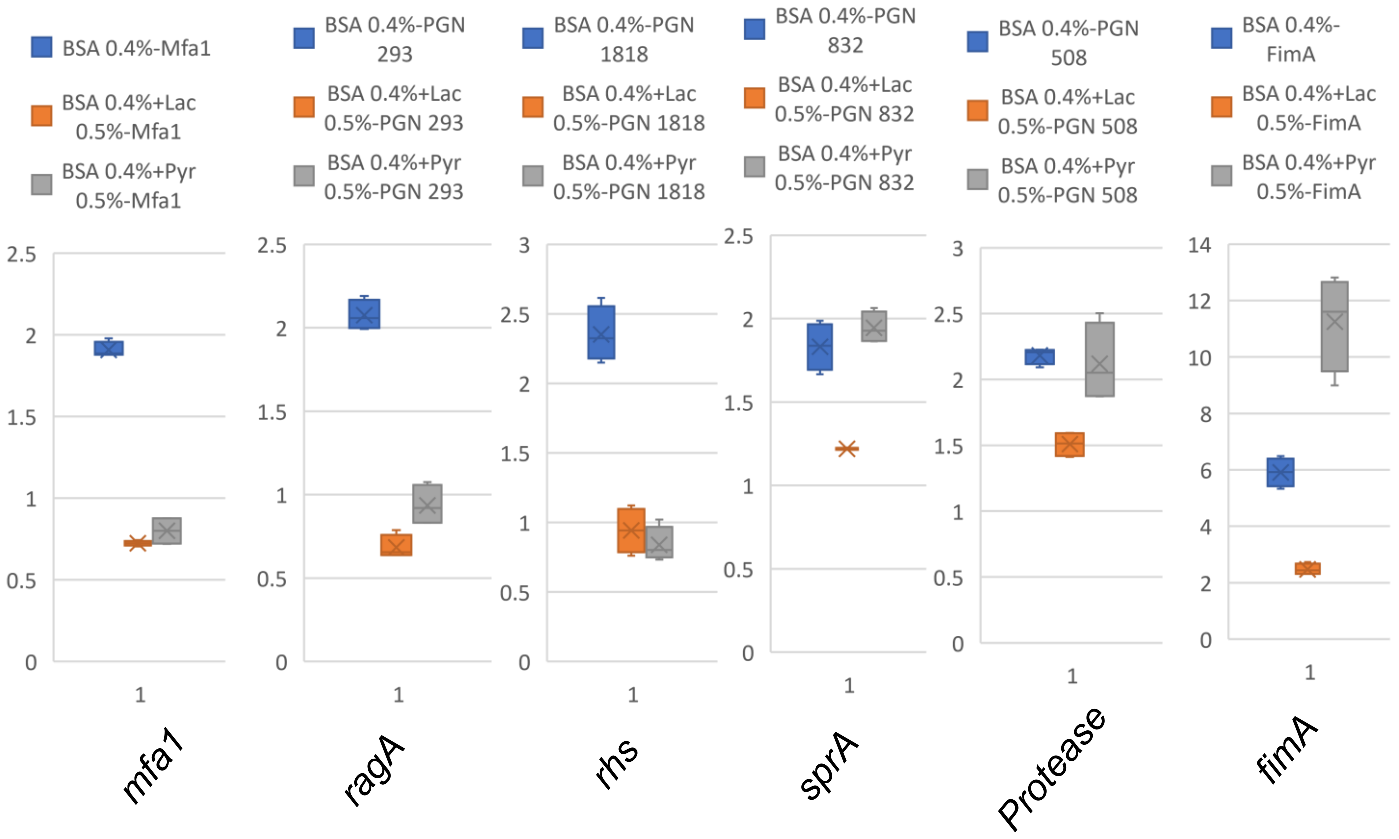

Box plot of Fig. 3A displaying the data distribution through their quartiles

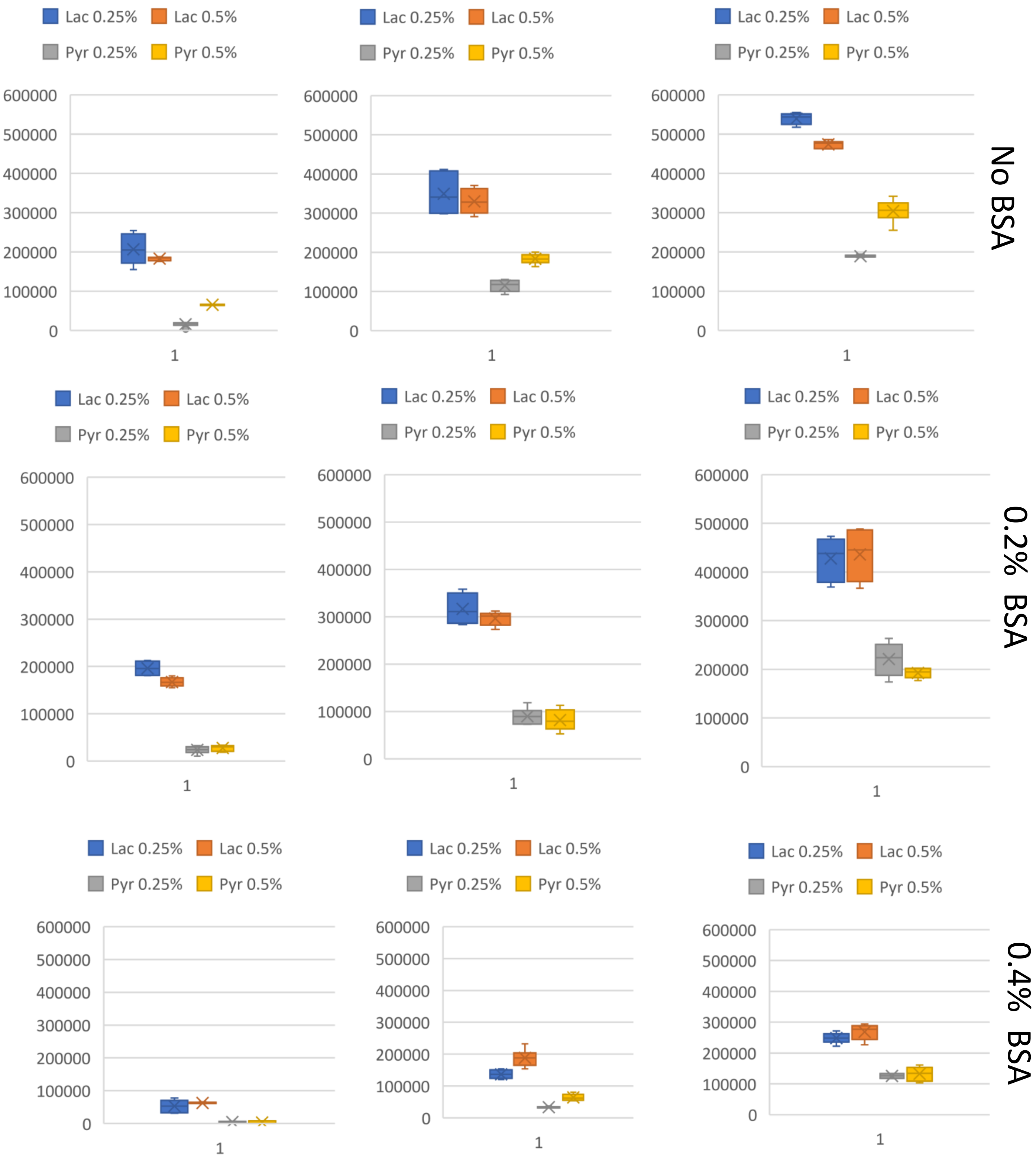

Box plot of Fig. 4A displaying the data distribution through their quartiles

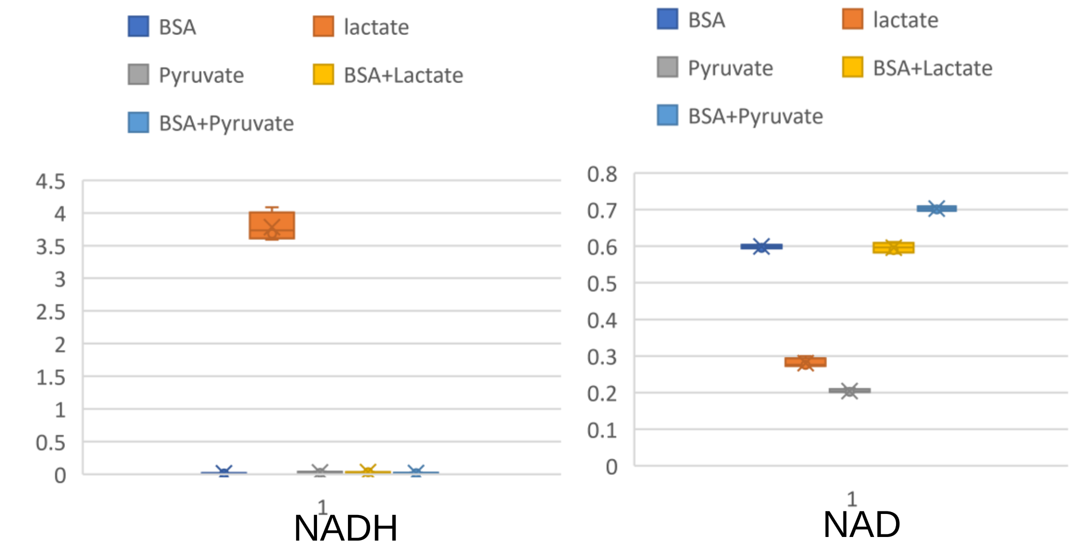

Box plot of Fig. 4B displaying the data distribution through their quartiles

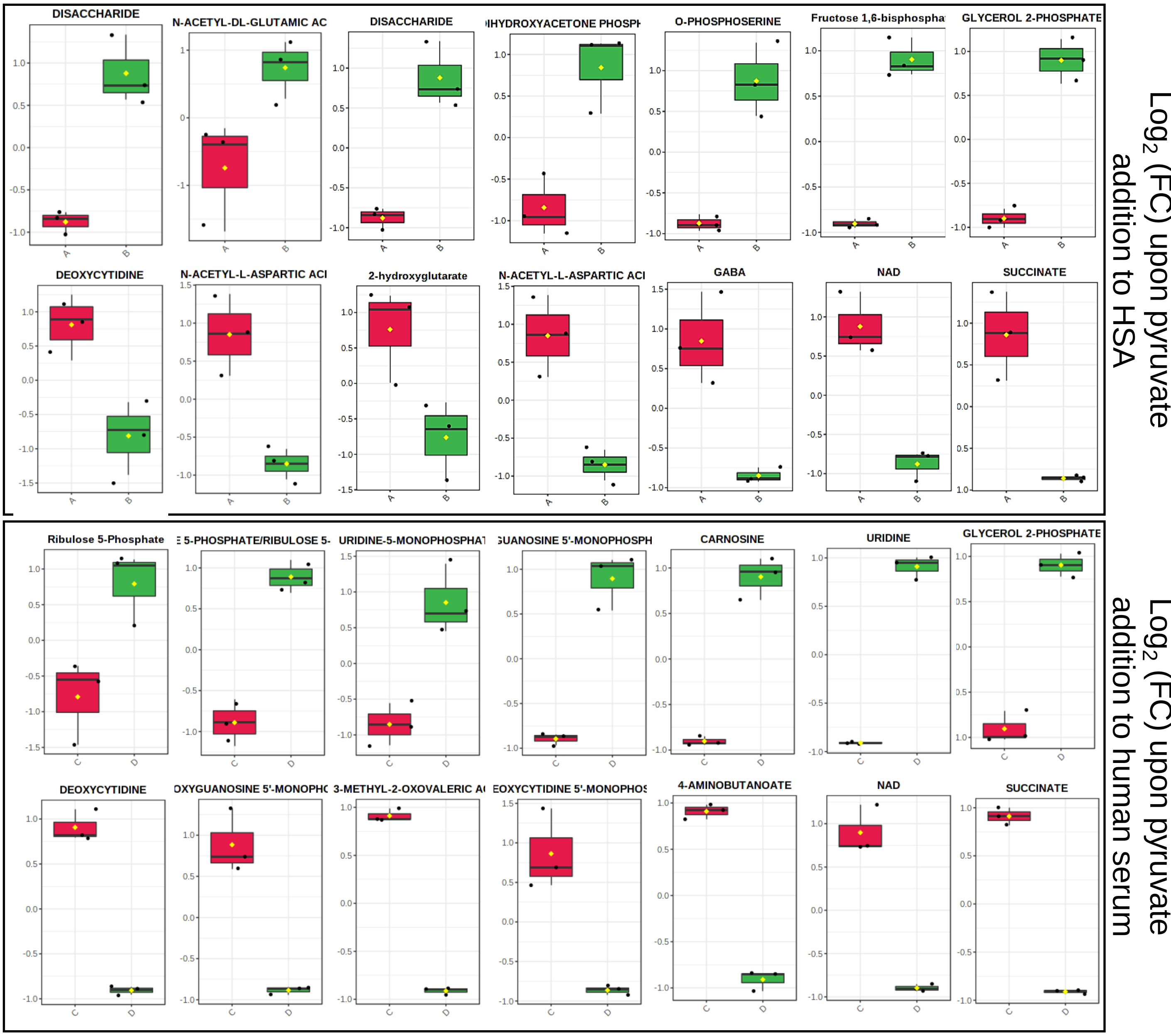

Box plot of Fig. 5 displaying the data distribution through their quartiles

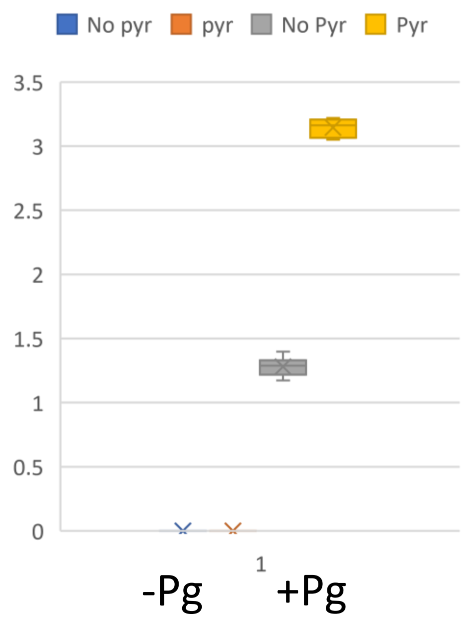

Box plot of Fig. 6A displaying the data distribution through their quartiles

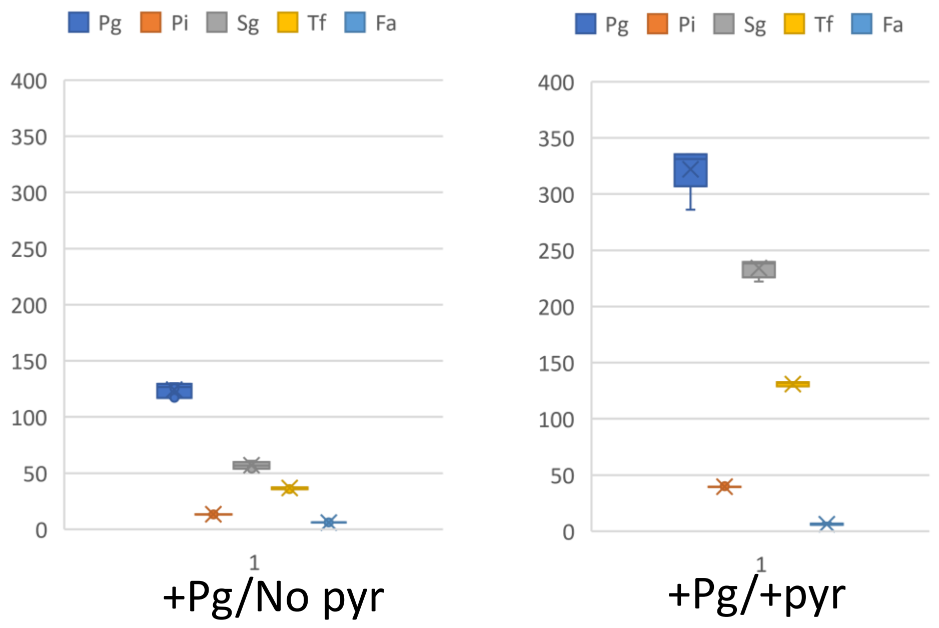

Box plot of Fig. 6B displaying the data distribution through their quartiles

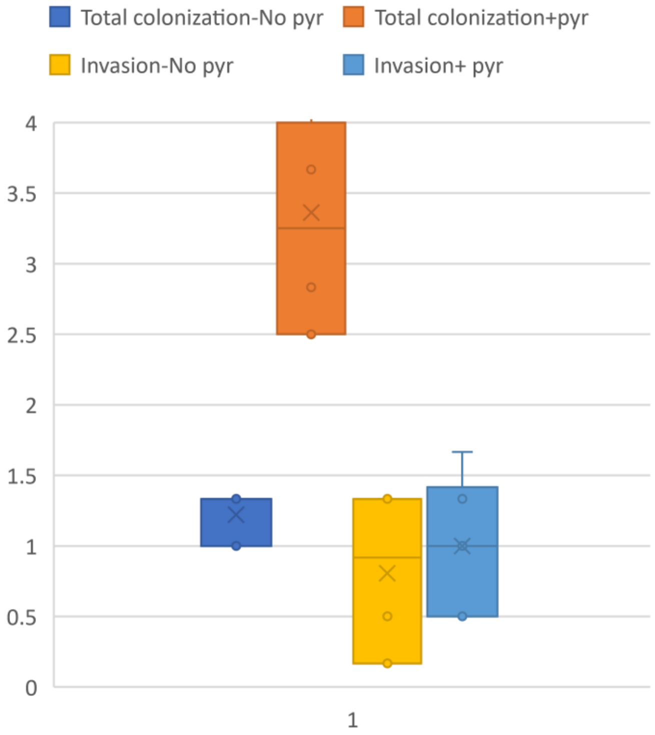

Anaerobic incubation

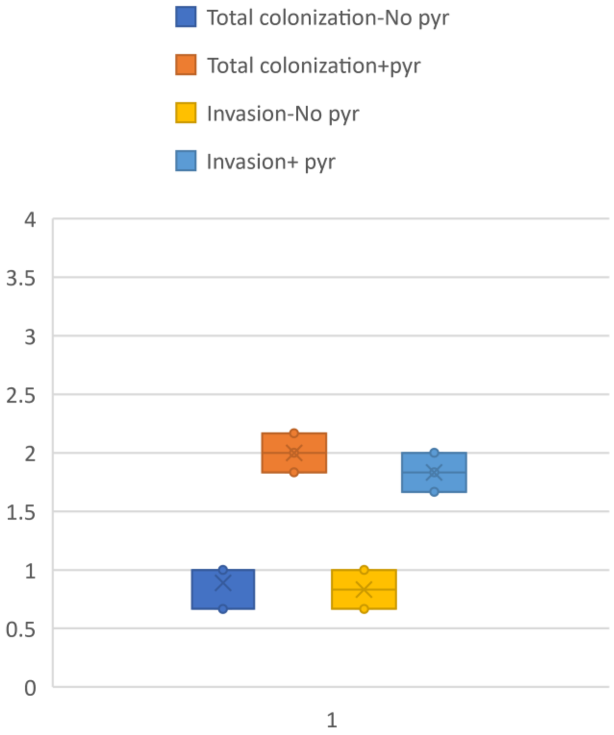

Aerobic incubation

Box plot of Fig. 6C and D displaying the data distribution through their quartiles

Supplementary Table 1- Identified metabolites in the metabolome of *P. gingivalis* cells grown in HSA or human serum, with and without adding pyruvate that show *p*-value < 0.05 in one-way ANOVA and *post hoc* analysis for multi-group analysis

Name	*p*-value
CARNOSINE	4.96E-12
URIDINE	2.67E-10
SUCCINATE	1.17E-09
UROCANATE	1.65E-09
ADENOSINE 3',5'-CYCLIC MONOPHOSPHATE	3.80E-09
N-ACETYLNEURAMINATE	3.84E-09
LL-2,6-DIAMINOHEPTANEDIOATE	6.22E-09
5'-DEOXYADENOSINE	1.58E-08
GLYCEROL 2-PHOSPHATE	2.46E-08
FRUCTOSE 1,6-BISPHOSPHATE	6.42E-08
GLYCERIC ACID	1.03E-07
L-ARGININE	1.43E-07
GLYCEROPHOSPHOCHOLINE	2.45E-07
4-AMINOBUTANOATE	2.48E-07
4-HYDROXYPHENYLACETATE	2.54E-07
O-PHOSPHOSERINE	2.57E-07
C12H22O11-DISACCHARIDE	2.90E-07
NAD	3.61E-07
L-CYSTEINE-S-SULFATE	7.87E-07
S-HEXYL-GLUTATHIONE	1.03E-06
TRYPTOPHAN	1.75E-06
DIHYDROXYACETONE PHOSPHATE	1.84E-06
ASPARAGINE	2.03E-06
L-HISTIDINE	2.13E-06
GUANOSINE 5'-MONOPHOSPHATE	2.36E-06
5-HYDROXYMETHYL-2-FURALDEHYDE	2.38E-06
4-OXOPROLINE	2.45E-06
AGMATINE SULFATE	3.58E-06
L-ISOLEUCINE	3.65E-06
ALPHA-KETOGLUTARIC ACID	3.73E-06
CITRULLINE	7.28E-06
N-ALPHA-ACETYL-L-LYSINE	7.52E-06
L-TYROSINE	8.84E-06
C6H12O6-HEXOSE/KETOSE/INOSITOL	9.01E-06
3-HYDROXYPHENYLACETATE	9.66E-06
L-GLUTAMIC ACID	9.86E-06
GLUCOSE/FRUCTOSE	1.08E-05
FUMARIC ACID	1.10E-05
CMP	1.40E-05
HEXOSE-6-PHOSPHATE	2.15E-05
PANTETHEINE	2.63E-05
N-METHYL-L-GLUTAMATE	3.10E-05
N-ACETYL-D-MANNOSAMINE	4.19E-05
4-HYDROXY-L-PHENYLGLYCINE	4.66E-05
N-ACETYL-L-ASPARTIC ACID	5.32E-05

N-AMIDINO-L-ASPARTATE	7.78E-05
5-OXO-L-PROLINE	8.11E-05
D-GLUCURONIC ACID/D-GLUCURONOLACTONE/D--GALACTURONIC ACID	8.61E-05
ASPARTATE	9.35E-05
N-ACETYL-HEXOSAMINE	0.000163
2-HYDROXYPHENYLALANINE	0.000165
NICOTINAMIDE MONONUCLEOTIDE	0.000165
DAMP	0.000173
SPERMIDINE	0.000175
L-VALINE	0.000178
TRYPTOPHAN-NH3	0.000191
HEXOSE-DISACCHARIDE	0.000209
D-RIBOSE 5-PHOSPHATE/RIBULOSE 5-PHOSPHATE	0.000246
DEOXYCYTIDINE	0.000248
C5-SUGAR ALCOHOL	0.000286
XANTHINE	0.000289
L-LYSINE	0.00033
URACIL	0.000341
L-ALLOTHREONINE	0.000375
PYRIDOXAL	0.000427
SARCOSINE/BETA-ALANINE	0.000468
L-CYSTINE	0.000498
N-BOC-L-TRYPTOPHAN	0.000525
NICOTINATE	0.000535
D-GLUCOSAMINE 6-PHOSPHATE	0.000537
ALPHA-AMINOADIPATE	0.000729
ISOCITRIC ACID	0.000819
METHIONINE SULFOXIDE	0.00088
THYMINE	0.000895
L-PROLINE	0.001076
BOC-D-PHENYLALANINE	0.001095
TRYPTOPHAN-2,3,3-D3	0.001175
L-ASPARAGINE	0.001489
SALICYLIC ACID-D4	0.001606
ORNITHINE	0.001722
GLUCOSAMINE/MANNOSAMINE	0.001812
L-ISOLEUCINE	0.00182
GLUCONIC ACID/D-GULONIC ACID GAMA-LACTONE	0.002042
5-HYDROXY-N-FORMYLKYNURENINE	0.003124
5-HYDROXY-L-TRYPTOPHAN	0.003461
GLUTARATE	0.003548
L-LEUCINE	0.003618
D-RIBOSE	0.00369
ALANINE	0.005109
NPAI-METHYL-L-HISTIDINE	0.007404
PHENYLALANINE	0.007782
N-FORMYL-L-METHIONINE	0.009608
GLYCYL-L-LEUCINE	0.009623

L-METHIONINE	0.011336
N-BOC-L-ASPARTIC ACID	0.015979
TRYPTOPHAN-2,3,3-D3-NH3	0.01787
ALDOPENTOSE	0.020202
3-SULFINO-L-ALANINE	0.020318
PYRIDOXAL 5'-PHOSPHATE	0.021224
FORMYLKYNURENINE	0.02263
ADENINE	0.025283
L-GLUTAMINE	0.029284
2'-DEOXYGUANOSINE 5'-MONOPHOSPHATE	0.031095

Supplementary Table 2- Identified metabolites in the metabolome of *P. gingivalis* cells grown in HSA or human serum with and without adding pyruvate that show Variable Importance in Projection (VIP) greater than 1.0 upon a multi-group analysis and PLS-DA model

**Name**	**PLSDA_VIP**		
	Comp. 1	Comp. 2	Comp. 3
NAD	1.5003	1.4733	1.4678
5-HYDROXY-L-TRYPTOPHAN	1.4992	1.4724	1.4658
L-GLUTAMIC ACID	1.4967	1.4692	1.4603
SPERMIDINE	1.4935	1.4637	1.4528
D-GLUCOSAMINE 6-PHOSPHATE	1.4757	1.4297	1.4182
GUANOSINE 5'-MONOPHOSPHATE	1.4627	1.4139	1.4065
AGMATINE SULFATE	1.4573	1.4084	1.3972
N-METHYL-L-GLUTAMATE	1.4555	1.406	1.3948
CMP	1.4523	1.403	1.394
N-ACETYLNEURAMINATE	1.4471	1.3992	1.388
L-HISTIDINE	1.4437	1.3956	1.3849
ALPHA-KETOGLUTARIC ACID	1.4392	1.3906	1.3816
DAMP	1.4372	1.3883	1.3783
5-OXO-L-PROLINE	1.4321	1.3836	1.3734
N-AMIDINO-L-ASPARTATE	1.4321	1.3848	1.3738
4-OXOPROLINE	1.4297	1.3817	1.3708
XANTHINE	1.4038	1.3985	1.3881
ETHYLMALONIC ACID	1.3989	1.3608	1.352
L-ASPARAGINE	1.3963	1.3491	1.3411
CITRULLINE	1.3952	1.3593	1.3571
ALPHA-AMINOADIPATE	1.3939	1.3466	1.3358
N-ALPHA-ACETYL-L-LYSINE	1.3815	1.3404	1.3299
NICOTINAMIDE MONONUCLEOTIDE	1.3766	1.3318	1.3215
FUMARIC ACID	1.3658	1.3256	1.315
N-FORMYL-L-METHIONINE	1.3658	1.3576	1.3475
GLUCONIC ACID/D-GULONIC ACID GAMA-LACTONE	1.3638	1.3176	1.3084
GLUTARATE	1.3617	1.3169	1.3072
L-VALINE	1.3594	1.3195	1.3118
L-LYSINE	1.3588	1.3132	1.3031
ISOCITRIC ACID	1.3568	1.3137	1.307
L-PROLINE	1.3433	1.2982	1.2932
LL-2,6-DIAMINOHEPTANEDIOATE	1.3427	1.3023	1.2984
3-HYDROXYPHENYLACETATE	1.321	1.2815	1.2718
S-HEXYL-GLUTATHIONE	1.3087	1.2697	1.2613
ASPARAGINE	1.3062	1.2744	1.2642
GLYCEROPHOSPHOCHOLINE	1.2991	1.2625	1.2543
ALANINE	1.2983	1.2707	1.2609
TRYPTOPHAN	1.2974	1.2628	1.2537
L-TYROSINE	1.2849	1.2474	1.2379

PANTETHEINE	1.2817	1.2408	1.2437
D-GLUCURONIC ACID/D-GLUCURONOLACTONE/D--GALACTURONIC ACID	1.2613	1.2328	1.2292
CARNOSINE	1.2585	1.2266	1.2257
2'-DEOXYGUANOSINE 5'-MONOPHOSPHATE	1.252	1.21	1.2005
L-ISOLEUCINE	1.2377	1.2246	1.2181
URIDINE	1.2295	1.1961	1.1973
TRYPTOPHAN-NH3	1.1941	1.167	1.1593
C5-SUGAR ALCOHOL	1.191	1.1516	1.1543
PYRIDOXAL 5'-PHOSPHATE	1.1892	1.1516	1.1423
DIHYDROXYACETONE PHOSPHATE	1.1827	1.1753	1.1691
2-HYDROXYPHENYLALANINE	1.1774	1.1518	1.1438
MALATE	1.1569	1.1186	1.1104
5-HYDROXY-N-FORMYLKYNURENINE	1.15	1.1158	1.113
D-RIBOSE	1.1395	1.1127	1.1146
CYTIDINE	1.1328	1.0953	1.0867
2'-DEOXYCYTIDINE 5'-MONOPHOSPHATE	1.1256	1.0965	1.0893
N-ACETYL-L-ASPARTIC ACID	1.1252	1.2294	1.228
L-CYSTEINE-S-SULFATE	1.1239	1.1279	1.1233
L-ARGININE	1.0907	1.1075	1.1004
4-HYDROXYPHENYLACETATE	1.0876	1.0706	1.0674
FORMYLKYNURENINE	1.0864	1.0579	1.0699
ADENINE	1.0807	1.044	1.0492
L-CYSTINE	1.0777	1.0411	1.0457
MGDG 20_70	1.0757	1.042	1.0496
DEOXYCYTIDINE	1.075	1.154	1.1449
ADENOSINE 5'-MONOPHOSPHATE	1.0655	1.0342	1.0259
4-HYDROXY-L-PHENYLGLYCINE	1.0652	1.0852	1.077
ISOCYTOSINE	1.0564	1.0212	1.0135
ALDOPENTOSE	1.0535	1.0243	1.0278
NPAI-METHYL-L-HISTIDINE	1.0447	1.0108	1.0191
URIDINE-5-MONOPHOSPHATE	1.0398	1.0203	1.0127
PYRIDOXAL	1.0327	1.0717	1.0665
C6H12O6-HEXOSE/KETOSE/INOSITOL	1.024	1.0329	1.0259
L-ALLOTHREONINE	0.9921	1.119	1.1107

Supplementary Table 3- Fold change of significant metabolites (*t* test, *p*-value < 0.05) in the biofilm cells upon concomitant utilization of pyruvate and HSA when compared with the HSA-only medium.

Name	FC	log2(FC)	raw.pval
HYPOXANTHINE	5.0905	2.3478	0.002155
FRUCTOSE 1,6-BISPHOSPHATE	2.6985	1.4322	0.000132
ADENOSINE 3',5'-CYCLIC MONOPHOSPHATE	4.7032	2.2336	0.00006201
GLYCEROL 2-PHOSPHATE	3.5493	1.8275	0.000351
L-ARGININE	1.548	0.6304	0.001268
5-HYDROXYMETHYL-2-FURALDEHYDE	1.6776	0.7464	0.001344
DISACCHARIDE	1.8325	0.87381	0.002103
NAD	0.51537	-0.95632	0.002156
O-PHOSPHOSERINE	2.6301	1.3951	0.002742
SUCCINATE	0.53556	-0.90088	0.004976
N-ACETYL-L-ASPARTIC ACID	0.41967	-1.2527	0.006874
GABA	0.43206	-1.2107	0.007464
DIHYDROXYACETONE PHOSPHATE	1.5044	0.58915	0.008792
5'-DEOXYADENOSINE	0.48017	-1.0584	0.008828
SPERMIDINE	0.6069	-0.72048	0.014408
DEOXYCYTIDINE	0.56216	-0.83096	0.01783
URIDINE	0.49116	-1.0257	0.022571
HEXOSE-6-PHOSPHATE	1.7881	0.8384	0.024922
HEXOSE-DISACCHARIDE	1.6245	0.69998	0.027357
D-RIBOSE 5-PHOSPHATE/RIBULOSE 5-PHOSPHATE	1.5028	0.58765	0.02825
N-ACETYL-HEXOSAMINE	1.5946	0.67322	0.029576
PYRIDOXINE	0.63007	-0.66642	0.032448
2-HYDROXYGLUTARATE	0.5115	-0.9672	0.038471
N-ACETYL-DL-GLUTAMIC ACID	1.6681	0.7382	0.049681
Fold change of significant metabolites (*t* test, *p*-value < 0.05) in the biofilm cells upon concomitant utilization of pyruvate and human serum when compared with the human serum-only medium.			
**NAME**	**FC**	**log2(FC)**	**raw.pval**
3-METHYL-2-OXOVALERIC ACID	0.19458	-2.3616	0.00000188
SUCCINATE	0.13912	-2.8456	0.00000423
URIDINE	8.9899	3.1683	0.0000126
UROCANATE	0.31556	-1.664	0.0000138
ADENOSINE 3',5'-CYCLIC MONOPHOSPHATE	3.9985	1.9995	0.00071524
4-AMINOBUTANOATE	0.2901	-1.7854	0.0000203
SPERMIDINE	0.23804	-2.0707	0.000029
MALATE	0.4271	-1.2274	0.0000313
L-GLUTAMIC ACID	0.4931	-1.0201	0.0000318
XANTHINE	0.4216	-1.2461	0.0000336
5'-DEOXYADENOSINE	0.26725	-1.9038	0.0000337
GLYCERIC ACID	0.26554	-1.913	0.0000418
THYMINE	0.2849	-1.8115	0.0000565
L-CYSTEINE	0.33837	-1.5633	0.000057

L-ISOLEUCINE	0.44137	-1.1799	0.0000628
DEOXYCYTIDINE	0.21622	-2.2094	0.0000651
L-ISOLEUCINE	0.48835	-1.034	0.000105
GLYCEROL 2-PHOSPHATE	3.3356	1.7379	0.00013
PHENYLALANINE	0.6316	-0.66291	0.000163
CARNOSINE	6.9762	2.8024	0.000195
LEU PRO	0.59605	-0.7465	0.000215
L-VALINE	0.34027	-1.5553	0.000236
ORNITHINE	0.46894	-1.0925	0.000255
ISOCYTOSINE	0.30474	-1.7143	0.00034
NAD	0.34531	-1.534	0.000393
HEXOSE-6-PHOSPHATE	3.3486	1.7436	0.000402
L-ALLOTHREONINE	0.45529	-1.1351	0.000405
LL-2,6-DIAMINOHEPTANEDIOATE	3.2599	1.7048	0.000519
GUANOSINE 5'-MONOPHOSPHATE	3.7606	1.911	0.000599
TRYPTOPHAN	0.59829	-0.74108	0.000612
4-HYDROXYPHENYLACETATE	0.42841	-1.2229	0.000706
D-RIBOSE 5-PHOSPHATE/RIBULOSE 5-PHOSPHATE	2.5095	1.3274	0.000893
C6H12O6-HEXOSE/KETOSE/INOSITOL	1.9894	0.99233	0.000965
N-METHYL-D-ASPARTIC ACID	0.53102	-0.91317	0.00097
N-AMIDINO-L-ASPARTATE	0.42812	-1.2239	0.001279
2'-DEOXYGUANOSINE 5'-MONOPHOSPHATE	0.35102	-1.5104	0.001445
AGMATINE SULFATE	0.39292	-1.3477	0.001636
DIHYDROXYACETONE PHOSPHATE	0.30507	-1.7128	0.001724
N-ACETYL-L-ASPARTIC ACID	0.40205	-1.3145	0.00188
GLUCOSE/FRUCTOSE	1.647	0.71987	0.002234
GLYCEROPHOSPHOCHOLINE	0.57286	-0.80375	0.002385
L-LEUCINE	0.55516	-0.84902	0.002708
5-HYDROXY-L-TRYPTOPHAN	0.56457	-0.82478	0.002819
N-ACETYL-D-MANNOSAMINE	0.34338	-1.5421	0.003238
PYRIDOXAL	1.8485	0.88638	0.003512
C5-SUGAR ALCOHOL	0.26395	-1.9217	0.003803
HEXOSE-DISACCHARIDE	2.4792	1.3099	0.004097
L-CYSTEINE-S-SULFATE	0.53499	-0.90241	0.004129
2'-DEOXYCYTIDINE 5'-MONOPHOSPHATE	0.34882	-1.5194	0.004296
URACIL	2.4986	1.3211	0.004311
ASPARTATE	0.43927	-1.1868	0.00637
URIDINE-5-MONOPHOSPHATE	2.3499	1.2326	0.006489
SARCOSINE/BETA-ALANINE	0.31463	-1.6683	0.007887
L-CYSTINE	0.35465	-1.4955	0.009222
2-HYDROXYPHENYLALANINE	0.62892	-0.66905	0.010491
S-HEXYL-GLUTATHIONE	0.58792	-0.76631	0.011168
C12H22O11-DISACCHARIDE	1.9253	0.94508	0.01167
ALANINE	0.46972	-1.0901	0.012598

D-RIBOSE	1.9454	0.96006	0.013941
D-GLUCURONIC ACID/D-GLUCURONOLACTONE/D--GALACTURONIC ACID	2.5444	1.3473	0.01566
L-GLUTAMINE	0.52277	-0.93575	0.016155
5-HYDROXYMETHYL-2-FURALDEHYDE	1.58	0.65989	0.016529
RIBULOSE 5-PHOSPHATE	1.6469	0.71978	0.025276
TRYPTOPHAN-NH3	0.55192	-0.85748	0.027777
3-SULFINO-L-ALANINE	0.52686	-0.9245	0.042352

**Supplementary Table 4**	
**Gene**	**RT-qPCR primers**
16sRNA	CCAAGTCGCGTGAAGGAAGA,q16sRNA.Fw
	CACCGCTGACTTACCGAACA,q16sRNA.Rv
fimA	TTCGGGGCAACACTCGTAAA,qFimA.Fw
	CCAAGTCGGCTCCGTTTTTC,qFimA.Rv
mfa1	GGAGAACGTCGCCAATACCT,qMfa1.Fw
	AATCAGCCAAAGTCGGCACT,qMfa1.Rv
sprA	ACTGACGCTGATTCGCAAGA,qPGN.832.Fw
	ACGGAAGCTGACACCGAAAT,qPGN.832.Rv
ragA	GGCTGAGTGACCTCCGATTC,qPGN.293.Fw
	CGAGAGCCTGGTGGTTGTAG,qPGN.293.Rv
rhs	CGTGGAAGAAACGCTGTCGG,qPGN.1818.Fw
	AATCCGTTTCCTCCGGCCTT,qPGN.1818.Rv
Cysteine protease	CGCTCAGCAAGAAGGAACGC,qPGN.508.Fw
	GAGCACCTTGGCCGGTACAT,qPGN.508.Rv
rgpA	TTCCTGACGGAGTGGCAGAC,qPGN.1970.Fw
	CGTAAGACTTGCCGTCAACGA,qPGN.1970.Rv
Na^+-NQR subunit (*nqrB*)	GCGGTTTGGTAGGAGCCTCA,qPGN.115.Fw
	CCCGGATTGAGCACTCGGAT,qPGN.115.Rv

**Supplementary Table 5-**16S rRNA gene primers used in quantification of individual population within multispecies biofilms		
**Species**	**16S rRNA gene primers**	
	**Forward**	**Reverse**
P. gingivalis 381	GGTGCGTAGGTTGTTCGGTA	TCCTGTTTGATACCCACGCC
S. gordonii DL1	ATTGCATCACTACCAGA	TGCTCGGTCAGACTTTCGTC
P. intermedia strain 17	CCATCAGGTTATGCTGGGCA	GTTGCAGACCTCAGTCCGAA
T. forsythia ATCC 43037	AGGATGACTGCCCTATGGGT	AAGCGACAAACTTTCACCGC
F. alocis ATCC 35896	ACCCTCAAGTTGCCAAAATTATTAT	TACTCCCTTTCTTCTGGTTAAATCT
